# Immunopathology and Immunosenescence, the Immunological Key Words of Severe COVID-19. Is There a Role for Stem Cell Transplantation?

**DOI:** 10.3389/fcell.2021.725606

**Published:** 2021-09-14

**Authors:** Mattia Emanuela Ligotti, Fanny Pojero, Giulia Accardi, Anna Aiello, Calogero Caruso, Giovanni Duro, Giuseppina Candore

**Affiliations:** ^1^Laboratory of Immunopathology and Immunosenescence, Department of Biomedicine, Neurosciences and Advanced Diagnostics, University of Palermo, Palermo, Italy; ^2^Institute for Biomedical Research and Innovation, National Research Council of Italy, Palermo, Italy; ^3^International Society on Aging and Disease, Fort Worth, TX, United States

**Keywords:** COVID-19, cytokine storm, immunopathology, immunosenescence, stem cell transplantation

## Abstract

The outcomes of Coronavirus disease-2019 (COVID-19) vary depending on the age, health status and sex of an individual, ranging from asymptomatic to lethal. From an immunologic viewpoint, the final severe lung damage observed in COVID-19 should be caused by cytokine storm, driven mainly by interleukin-6 and other pro-inflammatory cytokines. However, which immunopathogenic status precedes this “cytokine storm” and why the male older population is more severely affected, are currently unanswered questions. The aging of the immune system, i.e., immunosenescence, closely associated with a low-grade inflammatory status called “inflammageing,” should play a key role. The remodeling of both innate and adaptive immune response observed with aging can partly explain the age gradient in severity and mortality of COVID-19. This review discusses how aging impacts the immune response to the virus, focusing on possible strategies to rejuvenate the immune system with stem cell-based therapies. Indeed, due to immunomodulatory and anti-inflammatory properties, multipotent mesenchymal stem cells (MSCs) are a worth-considering option against COVID-19 adverse outcomes.

## Introduction

From December 2019 onwards, SARS coronavirus 2 (SARS-CoV-2) infection spread across the globe so rapidly that, on March 11, 2020, the World Health Organization (WHO) declared coronavirus disease-2019 (COVID-19) a global pandemic. While the pandemic is still in progress, the global scientific and medical community is focusing its resources on developing both anti-COVID-19 vaccines and therapeutic drugs, in order to gain control over the quick spread of this novel coronavirus.

SARS-CoV-2 is a *Betacoronavirus* of the *Coronaviridae* family that, like the other respiratory coronaviruses, is transmitted primarily via respiratory droplets, preferentially infecting lung alveolar epithelial cells using the human angiotensin-converting enzyme II (ACE2) as an entry receptor and the transmembrane serine protease 2 (TMPRSS2), which cleaves viral spike, for priming (Hoffmann et al., 2020; Wan et al., 2020).

This virus may infect people regardless of age, sex, and ethnicity but with different symptomatic presentation and outcomes. Indeed, clinical manifestations can range from being mild to severe respiratory disease, with a mean incubation period of approximately 5 days before first symptoms onset (Lauer et al., 2020; Li Q. et al., 2020; McAloon et al., 2020).

Fever, fatigue and dry cough are the most common symptoms in patients with mild or moderate disease. Severe characteristic symptoms include pneumonia, dyspnoea and subsequently acute respiratory distress syndrome (ARDS) that requires intensive care unit (ICU) admission with intubation and mechanical ventilation and can be fatal. In some cases, infected patients can present as either asymptomatic or paucisymptomatic, with little or no clinical manifestations (Baj et al., 2020; Chen N. et al., 2020; Gao Z. et al., 2021).

Although the majority of COVID-19 cases are mild symptomatic with a moderate case-fatality rate (Fu L. et al., 2020), older patients are at higher risk of getting severe COVID-19 disease, including hospitalization, and death (Wu and McGoogan, 2020). Last April, there were 140,322,903 confirmed COVID-19 cases and 3,003,794 deaths reported worldwide (World Health Organization, 2021), while in Italy, 3,870,131 total cases of COVID-19 and 116,927 deaths have been registered (Ministero della Salute, 2021). As of March 31, 2021, almost 86% of SARS-CoV-2 positive Italian patients that died were patients over 70 years old, with a mean age of 81 years and a median of 82 years (range 0–109), more than 30 years higher than that registered in infected people (median age 47; 51.2% females, 48.8% males) (Epicentro. Istituto Superiore di Sanità, 2021). Disease severity in older patients is due to not only the viral infection but also to host-related factors that make the individual particularly vulnerable to more severe outcomes of the infection.

An optimal model for identifying the host factors involved in susceptibility and/or protection to SARS-CoV-2 infection is represented by the centenarians, i.e., subjects who have reached ten or more decades of life, escaping the common age-related diseases. Their ability to repair damages and respond well to stressors is due to a combination of “positive features,” including intrinsic (genetic), extrinsic (environmental), and stochastic factors. Chance and life circumstances, nutrition, physical activity, and environmental exposures, pathogen burden and biology, with sex, genetic and epigenetic factors are all factors that may contribute to the longevity phenotype (Caruso, 2019). As a result, centenarians exhibit an optimal performance of the immune system, which contributes to their surprising tendency to recover from COVID-19 and complications (Lio et al., 2021). On the other hand, since the immune response to SARS-CoV-2 involves both innate and acquired immunity, dysregulation of the immune system has important consequences for the ability of the immune system to mount an effective response against the pathogen. Therefore, the remodeling of the immune response observed with aging can partly explain the age gradient in severity and mortality of COVID-19 (Cunha et al., 2020). In this context, the favorable prognosis and less severe signs on admission when hospitalized observed in young people may depend on an efficient acquired immune response against the virus (Gómez-Belda et al., 2021). On the other hand, the older immune system exhibits both cellular and humoral responses that are not efficient enough to limit infection and innate responses that are more prone to uncontrolled activation (Cunha et al., 2020). Various age-related quantitative and qualitative impairments have been reported in both innate and acquired immune responses, and they are commonly known as “immunosenescence.” Reduction in the ability to respond to new antigens, attributed to insufficient production of naïve immune cells, and increased memory responses, due to the lifelong antigen-exposure during persistent viral infections, have been associated with immunosenescence (Brunner et al., 2011; Aiello et al., 2019). With advancing age, there is gradual atrophy of the thymus, responsible for the development and selection of immunocompetent T cells (Kurd and Robey, 2016). This phenomenon, called thymic involution, involves structural changes in tissue mass and cellularity, as well as functional decline, ultimately resulting in a significantly decreased thymic output of naïve T cells, important for the response to novel pathogens that have not yet been encountered, contributing to shrinkage of the T cell diversity repertoire (Palmer, 2013). Since SARS-CoV-2 is a novel coronavirus with seemingly no prior sensitization, the naïve T cell decline caused by thymic involution might contribute to the increased vulnerability to severe COVID-19 outcomes in the older population, as well as reduced vaccination efficiency (Genebat et al., 2021; Wang et al., 2021).

Another characteristic of the older immune system is the presence of a chronic, low-grade inflammation, called inflammageing (Franceschi and Campisi, 2014), associated with increased production of pro-inflammatory cytokines, acute phase proteins, and oxidative stress (Fulop et al., 2018; Aiello et al., 2019). Inflammageing is considered one of the main risk factors for many age-related degenerative diseases and is caused by several factors, including life-long antigenic stimulation of the innate immune system by chronic infections, senescent cells and their senescence-associated secretory phenotype (SASP) (De Martinis et al., 2005; Hearps et al., 2012; Franceschi and Campisi, 2014). As a consequence, there is an increase in serum inflammatory mediators with age, including interleukin (IL)-6, tumor necrosis factor (TNF)-α, IL-1, and C-reactive protein (CRP) (Franceschi and Campisi, 2014; Barbé-Tuana et al., 2020). This inflammatory environment could exacerbate the immune response to the SARS-CoV-2. It has previously been seen that a dysregulated and excessive inflammatory response is responsible for sub-optimal T cells, antibody responses and impaired virus clearance which ultimately lead to worsening of SARS-CoV-1 and MERS-CoV infections, discovered in November 2002 and June 2012 respectively, with approximately 80 and 50% genetic similarity with SARS-CoV-2 respectively (To et al., 2013; Channappanavar and Perlman, 2017; Rabaan et al., 2020). Similarly, in SARS-CoV-2 infection, uncontrolled inflammation leads to severe and irreversible multi-organ damage, especially on the cardiac, hepatic and renal systems (Tian et al., 2020).

In the early phases of infection occurring in the respiratory tract, SARS-CoV-2 infected cells produce damage-associated molecular patterns (DAMP), such as ATP and nucleic acids, which are recognized by neighboring epithelial cells, endothelial cells and alveolar macrophages. This triggers the production of various pro-inflammatory cytokines and chemokines, allowing the recruitment of innate and acquired immune cells to the site of infection, including T cells (Tay et al., 2020; Ortega et al., 2021). In a healthy immune system, this initial inflammation recruits and activates virus-specific CD4+ and CD8+ T cells that kill the infected cells before the virus spreads, indirectly via induction of neutralizing antibody production o directly by inducing apoptosis, respectively. In a dysfunctional immune response, the accumulation of immune cells in the lungs can lead to the overproduction of pro-inflammatory cytokines, causing local cell and tissue damage with systemic implications (Tay et al., 2020). Considering that some cytokines can be both helpful in controlling the infection progression and harmful to the host, it is not easy to define a border between a regulated and a dysregulated response to a severe infection. Elevated levels of circulating cytokines, known as “cytokine storm,” were reported in COVID-19 patients, with variability in onset and duration in the early stages of infection but with convergent and often overlapping late-stage clinical manifestations (Fajgenbaum and June, 2020).

SARS-CoV-2 can infect also monocytes and macrophages through ACE2, by receptor-mediated endocytosis, and independently on ACE2, by pattern recognition receptors (PPRs), mechanisms. That results in their activation and secretion of large amounts of pro-inflammatory mediators, which contribute to local tissue and systemic inflammation (Jafarzadeh et al., 2020; Moore and June, 2020). Thus, in a scenario where the SARS-CoV-2 infection itself causes an excessive and uncontrolled systemic release of pro-inflammatory cytokines, the inflammageing process can aggravate this condition, amplifying inflammatory events and favoring the insurgence of ARDS, multiple organ failure, and finally death in severe COVID-19 older patients (Meftahi et al., 2020). For these reasons, it could be suggested that early identification of individuals at higher risk of virus-induced cytokine storm could help to determine the most effective treatment to prevent irreversible organ damage contributing to the relatively high mortality in older COVID-19 patients.

Also, it has been hypothesized that the accumulation of highly differentiated T cells with senescence-like features, together with inflammageing, may contribute to the destruction of lung tissue observed in severe COVID-19 patients (Akbar and Gilroy, 2020). Several studies have shown an expansion of senescent T cells in old individuals, driven by ongoing sub-clinical responses to chronic or persistent infections (Pawelec, 2018). These senescent T cells can express the natural killer cell receptors (NKRs) and can infiltrate into various tissues including the lung, acting as natural killer (NK) cells, thus promoting cytotoxicity against cells that expressed NKR ligands without prior antigen-specific priming (Pereira et al., 2020). According to this hypothesis, inflammation induced by SARS-CoV-2 infection in the lungs, hypothetically exacerbated by inflammatory monocytes infiltrated, would induce the expression of NKR ligands by epithelial cells of the host respiratory tract and lungs, and these may be recognized and killed by NK-like T cells (Akbar and Gilroy, 2020).

The severity of the disease, therefore, seems to depend primarily on the age and immune dysfunction. Nevertheless, these alone may still not be sufficient to explain the highest number of CFR in over 70 years population in South Korea, Spain, China, and Italy (Our World in data, 2021). Another important risk factor associated with the complications of COVID-19 is the presence of comorbidities, such as arterial hypertension, diabetes, and obesity, and older people are more likely to have these conditions (Guo L. et al., 2020; Li X. et al., 2020; Peña et al., 2020; Fresán et al., 2021). Many reports have confirmed the association between comorbidities and COVID-19 severity (Gao Y. D. et al., 2021), although it should be kept in mind that their high prevalence in the older population could be a confounding factor. The role of genetics is under scrutiny (Pojero et al., 2021).

In addition to all these risk factors, there is growing evidence of sex differences in severity and mortality of COVID-19. Specifically, when the fatality rate was stratified for gender, it was seen that males accounted for the majority of COVID-19 deaths with similar pieces of evidence in different countries (Alkhouli et al., 2020; Heras et al., 2020; Jin et al., 2020; Pradhan and Olsson, 2020; Sha et al., 2021). In Italy, the spread of COVID-19 has hit females (51.2%) slightly more than males (48.8%). However, data suggest that the mortality rate was higher for males than it was for females. Among all age groups, female Italian patients who died with complications associated with SARS-CoV-2 infection represented 43.9% and were older than men (median age: females 86 years, males 80 years). The mortality rate for female patients was 2.6%, while for male patients was 3.5% (Epicentro. Istituto Superiore di Sanità, 2021). This gender discrepancy of COVID-19–related morbidity and mortality can be attributed to a combination of biological sex differences, including differences in sex hormones involved in inflammatory processes and in expression levels of ACE2 and TMPRSS2, but literature data are not yet coherent (Gebhard et al., 2020; Haitao et al., 2020).

The role of sex hormones in immune responses has already been extensively investigated (Moulton, 2018; Márquez et al., 2020b; Fathi et al., 2021). Females show higher levels of estrogen and progesterone, with considerable fluctuations throughout the life span, while testosterone is more expressed in males, and these hormonal differences are assumed to play an important role in the innate and acquired immune responses (Fathi et al., 2021). For example, there is a large body of evidence for the role of estrogen in B cell development and activation and in various functions of T cells, in particular CD4+ T cells (T helper or TH) including differentiation, activation, cytokine production, homeostasis, and regulatory functions (Moulton, 2018). Estrogens have also been implicated in the homeostasis and activation of plasmacytoid dendritic cells (pDCs), key cells in antiviral immunity. Human female pDCs produce significantly higher interferon (IFN)-α levels in response to viral nucleic acids or Toll-like receptor (TLR) 7 agonists than pDCs derived from males, resulting in stronger secondary activation of CD8+ T cells (T cytotoxic or CTL) with impact on viral infection response (Berghöfer et al., 2006; Meier et al., 2009). The stronger responses observed in females result in faster pathogen clearance and a better response to vaccines, but could also be responsible for greater susceptibility to autoimmune diseases, considering that 80% of systemic autoimmune disorders occurring in females (Klein and Flanagan, 2016). In comparison to estrogens, several studies reported that exposure to androgens induces suppression of immune reactivity, affecting different immune cell subsets (Gubbels Bupp and Jorgensen, 2018). Detailed analysis on the sex differences in immune phenotype in SARS-CoV-2 infection has detected that, although plasma levels of many inflammatory cytokines and chemokines were generally elevated in patients, IL-8, IL-18, and CCL5 levels were significantly higher in male compared to female patients (Takahashi et al., 2020), suggesting a high risk of the cytokine storm.

It has also been proposed that the high plasma levels of ACE2 observed in males may partly explain their increased susceptibility to SARS-CoV-2 infection (Sama et al., 2020), although another study reported no differences in ACE2 expression in various human tissues between males and females or between young and old people (Li M. Y. et al., 2020). Similarly, an association between increased COVID-19 severity in males and expression of TMPRSS2, which is involved in cellular entry of the virus, has been theorized. TMPRSS2 gene expression is modulated by the androgen receptor signaling and increases following exposure to androgens (Lin et al., 1999). The upregulated TMPRSS2 expression in response to androgens could explain the sex-discrepancy in COVID-19 outcomes (Mjaess et al., 2020).

Therefore, immunosenescence, inflammageing (i.e., immunopathology), comorbidities, as well as male gender, play an important role in contributing to increased vulnerability to severe SARS-CoV-2 infection outcomes in older adults. Thus, the heterogeneity of patients makes it difficult to identify a consensus COVID-19 immune signature. In addition, some variability in the parameters used to classify the severity of COVID-19 could contribute to making it more difficult to compare the findings. However, understanding the immunological status prior to encountering the novel virus and how this is affected during infection provides interesting targets for better understanding the pathogenesis and treatment of SARS-CoV-2 infection.

In this review, we provide a detailed assessment of how aging impacts the immune response to the virus, providing when possible a parallelism with COVID-19-induced immune changes, focusing on possible strategies to rejuvenate the immune system with stem cell-based therapies. Indeed, due to immunomodulatory and anti-inflammatory properties, multipotent mesenchymal stem cells (MSCs) are a worth-considering option against COVID-19 adverse outcomes (Moradinasab et al., 2021).

## Cytokine Storm in COVID-19 Disease

Several analyses of the cytokine profile from COVID-19 patients have correlated the cytokine storm with lung tissue damage, multi-organ failure, and disease severity. Cellular origins of this large amount of cytokines is difficult to identify since SARS-CoV-2 infection requires the engagement of several immune cells, including the innate macrophages, dendritic cells, natural killer cells, and T and B lymphocytes (Ragab et al., 2020). It was reported significantly increased plasma levels of pro-inflammatory cytokines, including IL-1β, IL-6, IL-7, IL-8, IL-9, IL-10, interferon (IFN)-γ, and TNF-α in patients with COVID-19 than in healthy adults, but similar levels of IL-5, IL12p70, and IL-15. In addition, severe cases admitted to the ICU have shown higher plasma concentrations of IL-2, IL-7, IL-10, TNF-α, and other cytokines and chemokines than non-ICU patients (Huang et al., 2020). Levels of IL-6, mediator of the acute inflammatory response, and C-reactive protein (CRP), non-specific marker of inflammation, in these patients continue to increase over time and is significantly associated with ARDS and death in COVID-19 patients (Herold et al., 2020; Ruan et al., 2020; Wu et al., 2020; Zhou F. et al., 2020). Also analysis in bronchoalveolar lavage (BAL) fluid, a source of information of the microenvironment on bronchioles and lung alveoli, have shown higher levels of inflammatory cytokines, including IL-8, IL-6, and IL-1β, in severe cases compared to patients with moderate COVID-19 infection (Liao et al., 2020). When compared to survivor recovered patients, remarkably higher serum levels of IL-2R, IL-6, IL-8, IL-10, and TNF-α at admission were found in COVID-19 deceased patients, with a rapid increase during hospitalization, reinforcing the idea that dynamics of these cytokines and related receptors were highly associated with disease outcome (Cui et al., 2020). Likewise, it is reasonable to assume that the appropriate production of anti-inflammatory cytokines can counterbalance the systemic inflammation that occurs after infection. In male centenarians, it has been observed overexpression of anti-inflammatory variants in immune/inflammatory genes that could protect them from damaging effects of the cytokine storm associated with COVID-19 disease (Lio et al., 2021). Thus, cytokine storm prevention and control may be a crucial strategy in the treatment of COVID-19 patients. In line with that, it was proposed different anti-inflammatory strategies, in order to avoid or at least alleviate the cytokine storm. Among these, the use of IL-37 and IL-38 was suggested as potential therapeutic cytokines capable of inhibiting IL-1β and IL-6 (Conti et al., 2020).

## Innate Immunity: Natural Killer (NK) Cells

Natural killer cells are large granular lymphocytes, which play an important role in the host first line of defense against viral targets, via cell-mediated cytotoxicity mechanisms (CD56lowCD16+ subset) and secretion of pro-inflammatory cytokines (CD56hiCD16- subset). Among cytokines secreted by NK cells there are IFN-γ, with both antiviral and immune enhancing capabilities, TNF-α and granulocyte/macrophage colony-stimulating factor (GM-CSF) that can modulate the function of other innate and acquired immune cells (Mirandola et al., 2004; Paul and Lal, 2017). Thus, in addition to directly lysing the infected cell, NK cells are able to activate and mobilize other immune cells, including DCs, involved in the early stages of infection. Evidence of the critical role of NK cells in limiting viral infection prior to the induction of acquired immune responses has been provided by clinical studies of individuals who are deficient in NK cells and/or their functions, leading to current infections by viral pathogens (Orange, 2013). Likewise, changes in NK cell count, phenotype, and functions during aging may have a direct impact on COVID-19 disease progression.

Although less consistently than T cells (see below), NK cell numbers are reduced in COVID-19 patients, particularly evident in severe cases compared with mild disease patients and healthy controls (Jiang et al., 2020; Zheng M. et al., 2020; Belaid et al., 2021; Taghiloo et al., 2021), and their functional exhaustion was correlated with disease progression (Zheng M. et al., 2020). Single-cell analyses of bronchoalveolar samples of patients with COVID-19 have shown the presence of NK cells in the immune cell population analyzed, suggesting NK cell trafficking into the site of infection (Chua et al., 2020; Liao et al., 2020). Cytotoxic CD56low NK cells were depleted mainly in patients requiring mechanical ventilation, whereas cytokine-secreting CD56hi NK cells were significantly depleted in all COVID-19 patients (Wilk et al., 2020; Table 1).

**TABLE 1:** Observed fluctuation in NK cells in adult COVID-19 patients.

NK cell subsets	Observation	Analyzed patients	Compared group(s)	References
CD16+CD56+	Reduction (%)	Deceased	Survivors	Cui et al., 2020
	Reduction (#)	Deceased	Survivors	Cui et al., 2020; Belaid et al., 2021
	Reduction (#)	Pneumonia patients	HD	Wang F. et al., 2020
	No change (#)	Severe^η^	Mild	Wang F. et al., 2020
	Reduction (#)	Severe^η^	Mild^ζ^/moderate^θ^	Belaid et al., 2021
	Reduction (#)	Cases	HD	Jiang et al., 2020
CD16+	No change (%)	Cases	HD	Taghiloo et al., 2021
	Reduction (#)	Severe^η^	Mild^ζ^	Taghiloo et al., 2021
CD56+	No change (%)	Cases	HD	Taghiloo et al., 2021
	Reduction (#)	Severe^η^	Mild^ζ^	Taghiloo et al., 2021
CD56low	Reduction (%)	Critical^ι^	HD	Wilk et al., 2020
CD56hi	Reduction (%)	Cases	HD	Wilk et al., 2020
BAL NK cells	Reduction (%)	Severe^η^/Critical^ι^	Moderate^θ^	Liao et al., 2020
	Increase (%)	Moderate^θ^	HD	Liao et al., 2020

*NK cell subset, peripheral blood NK cell subset analyzed in the reported studies; BAL, bronchoalveolar lavage; (%), as frequency values; (#), as absolute number; Cases, COVID-19 patients (irrespective of severity); HD, healthy donors.*

*^ζ^Patients with clinical signs of pneumonia with one of the following: respiratory rate > 30 breaths/min, severe respiratory distress, or SpO_2_ < 90% on room air.*

*^η^Patients experiencing the following: respiratory failure, respiratory rate > 30 bpm, oxygen saturation < 93% at rest, arterial partial pressure of oxygen (PaO_2_)/fraction of inspired oxygen (FiO_2_) (PaO_2_/FiO_2_) ratio < 300 mmHg.*

*^θ^Fever, signs of airway disease, with or without a tomographic image indicating pneumonia.*

*^ι^Any of the following: requirement for mechanical ventilation, shock, or concomitant organ failure.*

Conversely, an increase in peripheral NK cell frequency and absolute cell count has been observed with advancing age, with a decreasing fraction and cytokines production of CD56hi NK cell subset and an expansion of CD56low NK cells (Solana et al., 2014; Gounder et al., 2018). Whether this correlates with disease severity in the older person is unclear.

Additionally, NK cell cytotoxic and secretory functions are tightly regulated by a dynamic balance between activating and inhibitory signals from an arsenal of membrane receptors, including killer cell immunoglobulin-like receptors (KIRs), randomly generated during NK cell differentiation and maturation (Vidal et al., 2011). Specific KIR/ligand interactions seem to be associated with COVID-19 disease severity as demonstrated in a very recent paper (Bernal et al., 2021) and by preliminary results from our ongoing study (unpublished observations).

## Other Cells of Innate Immunity: Dendritic Cells (DCs), Monocytes, Macrophages, Neutrophils, and Myeloid Derived Suppressor Cells (MDSCs)

In addition to NK cells, there are other important players of the innate immune system that provide the host’s first line of defense to the virus. Pattern recognition receptors (PRRs) expressed on innate immune cells, such as DCs and macrophages, recognize and bind surface viral epitopes, leading to anti-pathogen responses. The activation of these cells by the virus leads secretion of several inflammatory cytokines and chemokines, which activate and recruit other immune cells generating a positive feedback loop of inflammation (Pérez-Galarza et al., 2021). Aging alters also these actors involved in innate immunity in terms of both number/percentage and functionality, with different quantitative and qualitative consequences for DCs, monocytes, macrophages, neutrophils and myeloid derived suppressor cells (MDSCs) (Agrawal et al., 2017; Ventura et al., 2017; Oh et al., 2019; Feng et al., 2021). These changes in immune response may have an important impact in first phases of viral replication. In the following sections, we will discuss specific alterations in the other innate immunity cells in COVID-19 patients recalling those observed in the older immune system.

Although a clear difference in terms of severity and mortality was demonstrated comparing female and male COVID-19 patients as anticipated, to the best of our knowledge no study deeply explored sex related differences in SARS-CoV-2 infection as regards innate immunity.

### Dendritic Cells (DCs)

Dendritic cells are important immune cells, specialized to uptake, process and present antigen to naïve T cells, thus linking innate and acquired immunity. After pathogen internalization through recognition by PRRs, including TLRs, DCs process and present viral peptides to T cells via human leukocyte antigens (HLA)-T cell receptor (TCR) interaction, leading to activation of T cells.

Peripheral DCs are historically classified into three subsets based on their phenotype and functional characteristics: two subsets of myeloid DCs (mDCs) and one subset of plasmacytoid DCs (pDCs). The CD141+ mDC subset, named also myeloid (conventional) DC1 (cDC1), and CD1c+ mDCs, or cDC2, is both the primary source of IL-12, driving a TH1 response, but these cells are also able to secrete IL-6 and TNF-α. CD123+ pDCs, instead, produce high amounts of type I IFNs, critical for antiviral response (Collin et al., 2013; Mathan et al., 2013). An additional subset of DCs, called slanDCs, was identified by the use of M-DC8 monoclonal antibody, directed against the 6-sulfo LacNAc1 -slan- carbohydrate modification of *P*-selectin glycoprotein ligand-1. These cells are highly proficient in secreting inflammatory cytokines in both autoimmunity and infections (Schäkel et al., 1998; Dutertre et al., 2012; Günther et al., 2012; Hänsel et al., 2011, 2013; Tufa et al., 2014, 2016; Micheletti et al., 2016; Iannetta et al., 2019).

As regards the involvement of DCs in determining SARS-CoV-2 infection susceptibility and COVID-19 severity, experimental data demonstrated that TLR7 plays a critical role through viral single-stranded RNA recognition in the endosomal compartments of pDCs (Moreno-Eutimio et al., 2020; Onofrio et al., 2020; Kim and Shin, 2021; Gabriele et al., 2021). A clinical study showed that loss-of-function variants in X-linked TLR7 genes were associated with impaired type I and II IFN responses in young males, suggesting that the difference in TLR7 gene dosage between males and females could explain, at least in part, the predisposition of males to developing severe COVID-19 (van der Made et al., 2020).

Numerical changes in DC subsets were investigated as putative factors influencing anti-SARS-CoV-2 responses, and various authors demonstrated that DCs tend to decrease in COVID-19 patients vs. healthy controls, but in some reports, data also reached a statistical significance comparing more severe cases with patients with a milder form of the disease (Matic et al., 2020; Zhou R. et al., 2020; Buttenschön and Mattner, 2021; Kvedaraite et al., 2021; Zingaropoli et al., 2021; Table 2).

**TABLE 2:** Observed fluctuation in DCs and DC subsets in adult COVID-19 patients.

DC type	Observation	Analyzed patients	Compared group(s)	References
DCs	Reduction (%)	Hospitalized	HD	Zhou R. et al., 2020
	Reduction (%)	Convalescent	HD	Zhou R. et al., 2020
CD11c+ cDCs	Reduction (%)	Hospitalized	Convalescent	Zhou R. et al., 2020
	Reduction (%)^/^	Severe/Critical^+^	HD	Matic et al., 2020
	Reduction (%)^/^	Severe/Critical^+^	Mild/Moderate^&^	Matic et al., 2020
	No change (%)	Mild^$^	Convalescent	Neeland et al., 2021
	Increase (%)	Mild/Moderate^&^	HD	Matic et al., 2020
CD123-CD11c+ myeloid DCs	Reduction (%)	Pneumonia	HD	Zingaropoli et al., 2021
	Reduction (%)	ARDS	non-ARDS	Zingaropoli et al., 2021
CD11c+ slanDCs	Reduction (%)	Pneumonia	HD	Zingaropoli et al., 2021
AXL+SIGLEC6+ pre-DCs	Reduction (#)	Cases	HD	Kvedaraite et al., 2021
AXL+CD1c+ pre-DC2	Reduction (#)	Cases	HD	Kvedaraite et al., 2021
CLEC9A+ cDC1	Reduction (#)	Cases	HD	Kvedaraite et al., 2021
CLEC9A- cDC2*	Reduction (#)	Cases	HD	Kvedaraite et al., 2021
CD163-CD14- DC3	Reduction (#)	Cases	HD	Kvedaraite et al., 2021
CD163-CD14+ DC3	Reduction (#)	Cases	HD	Kvedaraite et al., 2021
CD163+CD14+ DC3	Reduction (#)	Cases	HD	Kvedaraite et al., 2021
CD5+DC2	Reduction (%)	Severe^§^	Moderate^@^	Kvedaraite et al., 2021
CD163-CD14- DC3	Reduction (%)	Severe^§^	Moderate^@^	Kvedaraite et al., 2021
AXL+CD1c+ pre-DC2	Increase (%)	Severe^§^	HD	Kvedaraite et al., 2021
CD123+CD11c- pDCs	Reduction (#)	Cases	HD	Kvedaraite et al., 2021
	Reduction (%)	ARDS	non-ARDS	Zingaropoli et al., 2021
	Reduction (%)	Pneumonia	HD	Zingaropoli et al., 2021
CD123+Lyn- pDCs	Reduction (%)^/^	Severe/Critical^+^	HD	Matic et al., 2020
	Reduction (%)^/^	Severe/Critical^+^	Mild/Moderate^&^	Matic et al., 2020
	Reduction (%)	Mild/Moderate^&^	HD	Matic et al., 2020

*DC, dendritic cell; cDCs, conventional dendritic cells; pDCs, plasmacytoid dendritic cells; (%), as frequency values; (#), as absolute number; HD, healthy donors; ARDS, acute respiratory distress syndrome; Cases, COVID-19 patients (irrespective of severity).*

*^/^No statistically significant data provided.*

*^+^Fever or suspected respiratory infection with compromised respiratory function and worsening of respiratory symptoms with the necessity for mechanical ventilation.*

^&^Mild clinical symptoms of upper respiratory tract viral infection and signs of pneumonia without need for supplemental oxygen. *Including CD5+ DC2 and CD5– DC3.

*^@^Oxygen saturation -SO_2_- between 90 and 94% or 0.5 to 3 L/min oxygen requirement at screening.*

*^§^Treated at the intensive care unit -ICU- or at a high-dependency unit.*

*^$^Including coryza, headaches, nausea, fever, cough, sore throat, malaise, headaches, and muscle aches.*

A more detailed analysis of CD11c+ DCs accumulation/decrease as a function of both age and severity should be urgently performed in adult COVID-19 cases. In fact, non-plasmacytoid DCs are reported as numerically stable in the old people (Agrawal et al., 2017; Oh et al., 2019), but this trend seems to be reverted in severe COVID-19 patients, although an age specific stratification of the cases was not performed (Table 2; Matic et al., 2020; Zhou R. et al., 2020; Buttenschön and Mattner, 2021; Kvedaraite et al., 2021; Zingaropoli et al., 2021). Intriguingly, similar data were obtained comparing children in the acute phase of mild disease vs. their convalescent counterpart (Neeland et al., 2021), thus in the case of younger patients, milder disease is accompanied by CD11c+ DC fluctuations resembling those observed in adult severe cases. On the contrary, the contraction of CD11c+ DC pool was not documented comparing adult patients with mild symptoms vs. convalescent subjects (Neeland et al., 2021), and the same DC subset was even reported as increased in mild/moderate patients vs. healthy controls (Matic et al., 2020). Molecular mechanisms causing stability of CD11c+ DCs in older subjects seem to be compromised in both adults with severe disease and children with mild disease, and deserve further investigation.

Instead, plasmacytoid DCs tend to decrease in aging (Agrawal et al., 2017; Oh et al., 2019; Márquez et al., 2020b). A reduction in the plasmacytoid DCs was also reported in COVID-19 patients irrespective of severity (Table 2; Arunachalam et al., 2020; Matic et al., 2020; Kvedaraite et al., 2021; Zingaropoli et al., 2021).

During physiological aging processes, DCs exhibit multiple functional defects ranging from mitochondrial dysfunction to an impairment in antigen uptake/presentation and altered cytokine secretion (Giefing-Kröll et al., 2015; Agrawal et al., 2017; Ventura et al., 2017; Oh et al., 2019; Salminen et al., 2019). DCs showed some degree of immunophenotypic/functional alteration also in COVID-19. CD11c+ cDCs in hospitalized patients and convalescent (follow up) outpatients exhibited less surface expression of the co-stimulatory molecule CD86 than DCs in healthy subjects (Zhou R. et al., 2020; Buttenschön and Mattner, 2021). Similarly, expression of CD86 and HLA-DR was uniformly reduced on circulating DC precursor (pre-DC), pre-DC2, CD5+ DC2, CD163- CD14- DC3, CD163- CD14+ DC3, and CD163+ CD14+ DC3 in ICU or high dependency unit severe patients vs. healthy donors (Kvedaraite et al., 2021). Also, cDCs in both hospitalized patients and convalescent (follow up) outpatients showed resistance toward maturation stimuli, and an impaired ability to produce type I IFNs which was more prominent in hospitalized patients (Zhou R. et al., 2020; Buttenschön and Mattner, 2021), recalling a similar panel of IFN secretion defects documented in older individuals (Agrawal et al., 2017). Instead, the ability to answer to type I IFN signaling seemed to be preserved in COVID-19 patients, as demonstrated by the expansion of cell surface receptor tyrosine kinase (AXL) expressing fraction among DC1 pool and the reduced expression of c-kit as measured in DC1 (Rhodes et al., 2019; Kvedaraite et al., 2021). INF-signaling was also highly activated in BAL DC1 (Kvedaraite et al., 2021). However, myeloid DCs showed an impaired ability to answer to TLR stimulation (Arunachalam et al., 2020).

Activation of peripheral blood plasmacytoid DCs was made evident by the decreased expression of CD45RA in COVID-19 patients vs. healthy controls (Kvedaraite et al., 2021). Similarly, a reactive response in COVID-19 patients BAL plasmacytoid DCs was demonstrated by activation of cytokine and chemokine signaling pathways at a transcriptional level (Kvedaraite et al., 2021). However, another report documented an impairment in plasmacytoid DC function, with a reduced production of IFN-α and TNF-α after TLR stimulation (Arunachalam et al., 2020). Plasmacytoid DCs showed less HLA-DR in ICU or high dependency unit severe patients than in moderate cases (Kvedaraite et al., 2021).

### Monocytes and Macrophages

Alterations of monocyte phagocytic and cytokine secreting functions were consistently documented in older individuals; however, numerical fluctuations do not affect all monocyte subsets. Based on the expression of the surface markers CD14 and CD16, circulating monocyte can be dived into three subsets: classical, intermediate, and non-classical monocytes, with CD14 decreasing from classical to non-classical subsets, and CD16 following an opposite expression pattern (Ziegler-Heitbrock et al., 2010).

CD14+ CD16– classical monocyte numbers do not change during aging, whereas CD14+ CD16+ non-classical monocytes tend to increase with age (Ventura et al., 2017; Oh et al., 2019; Feng et al., 2021). Despite these functional changes, monocytes seem to be key determinants of sex associated differences in immunosenescence, with a male superior ability in mounting inflammatory responses. In fact, men exhibit more chromatin accessibility at monocyte specific *loci* than women (Márquez et al., 2020a).

A sex specific study demonstrated that both male and female non-ICU patients exhibited a higher percentage of monocytes than male and female controls, respectively (Takahashi et al., 2020). While CD14+ CD16– classical monocyte frequency was overlapping among groups, CD14lowCD16- non-classical monocytes were detected with higher frequencies in non-ICU male patients vs. both male controls and female patients (Takahashi et al., 2020). However, such a gender related effect on non-classical monocytes was lost in all the studies pooling male and female patients and controls together, leading to inconsistent results ranging from the absence of observed changes in monocyte frequency irrespective of severity to a statistically significant reduction in the case of more serious symptoms (Table 3). In addition, to explain the irreproducibility of results for both classical and non-classical monocytes, age dependent repercussions on monocyte dynamics in COVID-19 should be taken into account in the case of severity-based studies. In fact, lower percentages of both CD14+CD16– classical and CD14lowCD16+ non-classical monocytes were encountered in peripheral blood of infected children experiencing mild symptoms vs. convalescent children sampled 4–7 weeks following test results (Neeland et al., 2021).

**TABLE 3:** Observed fluctuation in monocytic subsets in adult COVID-19 patients.

Monocytes	Observation	Analyzed patients	Compared group(s)	References
Classical CD14++CD16- monocytes	Reduction (%)	Hospitalized	HD	Zhou R. et al., 2020
	Reduction (%)	Convalescent	HD	Zhou R. et al., 2020
	No change (%)	Pneumonia	HD	Zingaropoli et al., 2021
	No change (%)	Mild^$^	Convalescent	Neeland et al., 2021
Classical CD14+CD16- monocytes	No change (%)	Severe^&^	Mild*	Carsetti et al., 2020
	No change (%)	Severe^&^	Asymptomatic	Carsetti et al., 2020
	No change (%)	Severe^&^	Contacts^+^	Carsetti et al., 2020
	No change (%)	Mild*	Asymptomatic	Carsetti et al., 2020
	No change (%)	Mild*	Contacts^+^	Carsetti et al., 2020
	No change (%)	Asymptomatic	Contacts^+^	Carsetti et al., 2020
CD14+CD16+ non-classical monocytes	Reduction (%)	Pneumonia	HD	Zingaropoli et al., 2021
	Reduction (%)	ARDS	Non-ARDS	Zingaropoli et al., 2021
CD14-CD16+ non-classical monocytes	Reduction (%)	Severe^&^	Mild*	Carsetti et al., 2020
	Reduction (%)	Severe^&^	Asymptomatic	Carsetti et al., 2020
	Reduction (%)	Severe^&^	Contacts	Carsetti et al., 2020
	No change (%)	Mild*	Asymptomatic	Carsetti et al., 2020
	No change (%)	Mild*	Contacts^+^	Carsetti et al., 2020
	No change (%)	Asymptomatic	Contacts^+^	Carsetti et al., 2020
CD14lowCD16+ non-classical monocytes	Reduction (%)	Mild^$^	Convalescent	Neeland et al., 2021
CD14lowCD16++ non-classical monocytes	Reduction (%)	Severe^§^	HD	Kvedaraite et al., 2021
CD14++CD16+ intermediate monocytes	Reduction (%)	Pneumonia	HD	Zingaropoli et al., 2021
CD14+CD16+ intermediate monocytes	Increase (%)	Moderate^@^	HD	Kvedaraite et al., 2021
CD14+CD16+ intermediate monocytes	Increase (%)	Severe^§^	HD	Kvedaraite et al., 2021
CD14+CD16+/– intermediate monocytes	No change (%)	Severe^&^	Mild*	Carsetti et al., 2020
	No change (%)	Severe^&^	Asymptomatic	Carsetti et al., 2020
	No change (%)	Severe^&^	Contacts^+^	Carsetti et al., 2020
	No change (%)	Mild*	Asymptomatic	Carsetti et al., 2020
	No change (%)	Mild*	Contacts^+^	Carsetti et al., 2020
	No change (%)	Asymptomatic	Contacts^+^	Carsetti et al., 2020

*(%), as frequency values; HD, healthy donors; ARDS, acute respiratory distress syndrome, asymptomatic patients who tested positive but had no symptoms.*

*^$^Including coryza, headaches, nausea, fever, cough, sore throat, malaise, headaches, and muscle aches.*

*^§^Treated at the intensive care unit -ICU- or at a high-dependency unit.*

*^@^Oxygen saturation -SO2- between 90 and 94% or 0.5 to 3 L/min oxygen requirement at screening.*

^&^Pneumonia (fever, cough, dyspnoea, fast breathing) plus one of the following: respiratory rate > 30 breaths/min; severe respiratory distress; or SpO_2_ < 90% on room air. *Patients requiring no hospitalization and experiencing symptoms like with fever, myalgia, and fatigue without obvious chest high resolution computed tomography findings for COVID-19.

*^+^Contacts of SARS-CoV-2 confirmed cases who were negative by qPCR.*

The observed discrepancy among the available reports on classical and non-classical monocytes may also be a consequence of the lack of shared criteria to stratify SARS-CoV-2 infected subjects according to severity (Table 3; Wilk et al., 2020; Zhou R. et al., 2020; Kvedaraite et al., 2021; Neeland et al., 2021; Zingaropoli et al., 2021). In addition, from a more technical point of view, small differences in the immunophenotypic parameters evaluated to define monocytic subsets may be appreciated and may account for the lack of reproducibility of the observed data (Table 3).

Data about intermediate monocytes are also conflicting, requiring the inclusion of both gender- and age-related effects on disease severity in the study design. Percentage of CD14+CD16+ intermediate monocytes were reported as augmented in both male and female non-ICU patients vs. their sex matched controls, with more statistically significant results obtained for female subjects (Takahashi et al., 2020). On the contrary, a reduction in the frequency of peripheral blood CD14+CD16+ intermediate monocytes was detected in children with mild disease vs convalescent children sampled (Neeland et al., 2021). As reported in Table 3, a lower frequency of CD14++CD16+ intermediate monocytes was demonstrated in a cohort of patients with COVID-19 pneumonia vs. healthy donors (Zingaropoli et al., 2021). Instead, CD14+CD16+ intermediate monocytes were higher in both moderate and severe cases vs. healthy controls (Kvedaraite et al., 2021). No statistically significant difference was detected comparing contacts of SARS-CoV-2 cases with severe, mild and asymptomatic patients (Carsetti et al., 2020; Table 3). In none of these reports, a sex and age specific analysis was performed (Carsetti et al., 2020; Kvedaraite et al., 2021; Zingaropoli et al., 2021).

The effect of sex and age on COVID-19 patients’ monocyte function was not dissected, although some preliminary data explored the behavior of monocyte subsets according to severity. As regards monocyte functionality, levels of CD14+CD16- classical and CD14+CD16+ intermediate monocyte HLA-DR and CD86 were reduced in ICU or high-dependency unit patients vs. both healthy controls and moderate cases of the disease, whereas the same markers were reduced on CD14lowCD16++ non-classical monocytes in severe patients vs. subjects with moderate disease only (Kvedaraite et al., 2021). Similar piece of evidence as regards HLA-DR expression emerged in another study (Arunachalam et al., 2020). Moreover, as previously observed for the mDCs, also COVID-19 patients CD14+ monocytes showed an impaired ability to answer to TLR stimulation, reinforcing the idea of an impaired innate response during COVID-19 disease (Arunachalam et al., 2020). However, classical monocytes from severe (according to the National Early Warning Score) COVID-19 cases showed type I IFN response, and exacerbation of TNF/IL-1β-driven inflammation (Lee et al., 2020; Kim C.W. et al., 2021). Consistently, in the lung environment, both BAL macrophages from severe patients (Liao et al., 2020; Kim C.W. et al., 2021) and airway macrophages from critical (according to WHO guidelines) cases (Chua et al., 2020) exhibited proinflammatory characteristics (Chua et al., 2020; Liao et al., 2020; Kim C.W. et al., 2021).

### Neutrophils

The neutrophil number is preserved during aging, but the reduction in CD16 expression accounts for a notable functional decline (Ventura et al., 2017; Oh et al., 2019; Salminen et al., 2019; Cunha et al., 2020; Zimmermann and Curtis, 2020). Also, using animal models it was documented that lung neutrophils exhibit signs of exhaustion due to exposure to inflammatory mediators associated with advancing age (Ventura et al., 2017; Domingues et al., 2020).

Analysis of peripheral blood composition in COVID-19 cases revealed that in ICU patients neutrophil count was higher than that observed in non-ICU patients (Wang D. et al., 2020), and that neutrophil count was higher in deceased patients vs. survivors (Du et al., 2020; Fu Y. Q. et al., 2020).

SSChiCD16+ low density neutrophils, including migrating cells and immature elements in both healthy conditions and inflammation (Silvestre-Roig et al., 2019; Hassani et al., 2020), seem to exert a protective role in both adults and children. In fact, their accumulation was documented in PCR-negative adults and children exposed to SARS-CoV-2 in the household, with higher percentages recorded when sampling happened during the convalescence period (up to 7 weeks after positive test results) vs. exposure during the acute phase (Neeland et al., 2021).

No changes were recorded for neutrophils and activated CD63+ neutrophils comparing both adult SARS-CoV-2 positive and adult SARS-CoV-2 exposed subjects in acute and convalescent phase (Neeland et al., 2021). Instead, an increase in the percentage of CD63+ neutrophils was detected comparing infected children experiencing mild symptoms with both convalescent children sampled 4–7 weeks following test results and PCR negative children exposed to SARS-CoV-2 through household close contact with a positive in the acute phase (Neeland et al., 2021). Since expression of CD63 on neutrophils is a sign of activation (Silvestre-Roig et al., 2019), it would be worth deepening if more older individuals may experience a difference in neutrophil degranulation in comparison with younger patients, and if such a difference may be related to COVID-19 severity.

### Myeloid Derived Suppressor Cells (MDSCs)

Myeloid-derived suppressor cells (MDSCs) are cells of the myeloid lineage exerting a suppressive function on other immune cells through numerous molecular mechanisms (Salminen et al., 2019). MDSCs can be divided into two main branches, CD11b+CD33+CD15+CD14-HLA-DR- granulocytic MDSCs and CD11b+CD33+CD15-CD14+HLA-DRlo/- monocytic MDSCs, together with a further subset devoid of both granulocytic and monocytic markers (Salminen et al., 2019; Bergenfelz and Leandersson, 2020; Kramer and Abrams, 2020). Only the proportion of granulocytic MDSCs is reported as increased during aging (Salminen et al., 2019). However, to the best of our knowledge, no sexual dimorphism was described about distribution and function of MDSCs in individuals.

Expansion of functional granulocytic MDSCs was documented in COVID-19 patients vs. healthy donors, and was particularly evident in severe cases (Agrati et al., 2020) and in ICU patients (Sacchi et al., 2020). The percentage of monocytic myeloid derived suppressor cells (M-MDSCs) was higher in hospitalized patients vs. both convalescent (follow up) outpatients and healthy donors (Zhou R. et al., 2020; Buttenschön and Mattner, 2021).

## Acquired Immunity: T Cells

Among components of both innate and adaptive immune system, there seems to be more coherence in the information on T cell responses observed during COVID-19 disease. A central object of study in several reports, the information obtained to date on changes in cellular immunity, and discussed below in relation to age and sex, may help us to better understand the differences in infection outcomes observed.

Functionally, T cells are considered naïve until they encounter their cognate antigen in the periphery, and phenotypically they are CCR7+CD45RA+CD28+CD27+ cells. In the context of SARS-CoV-2 infection, after encountering novel viral antigens, naïve T cells expand and differentiate into different types of effector cells, whose protective roles encompass TH-cell-mediated activation of B cells to produce virus-specific antibodies and elimination of virus-infected cells by CTLs. SARS-CoV-2-specific T cells have been reported in COVID-19 patients (Braun et al., 2020; Sekine et al., 2020). Following activation, the chemokine receptor CXCR3 is rapidly induced on naïve cells T cells and preferentially remains highly expressed on TH1 cells and effector CD8+ T cells (Groom and Luster, 2011). In contrast to innate immune cells, T cells develop a memory for repeated challenges. Indeed, following clearance of pathogen, most of effector T cells die but a small subset further differentiates into long-lived memory T cells that provide long-term protective immunity (Mahnke et al., 2013). So, in the event of a second contact with the virus, memory T cells rapidly mature into effector cells, responding to infection. Previously studies in mice have highlighted the importance of specific cell-mediated memory in protection from SARS-CoV-1 (Zhao J. et al., 2010; Channappanavar et al., 2014). It was shown that in some individuals exposure to SARS-CoV-2 has induced virus-specific T cell responses even in the absence of seroconversion, suggesting that individuals with no detectable antibodies may nonetheless be protected by cellular immunity (Gallais et al., 2021). This raises the question of whether T-cell responses would be more sensitive indicators of SARS-Co-V-2 exposure than antibody quantification of anti-nucleoprotein and anti-spike IgG or IgA levels in peripheral blood. However, individual variability in immune response must be taken into account, especially in view of the profound immune changes observed with aging. Thus, a thorough understanding of the role of T cells in the immune response to SARS-CoV-2 and how aging may impair their responsiveness is essential to gain insights into both the individual ability to respond to the first infection and the quality, magnitude, and durability of protective immunity against reinfection with SARS-CoV-2.

As this is an emerging pathogen to which the human population has never previously been exposed, although cross-reactive T cell recognition between circulating ‘common cold’ coronaviruses and SARS-CoV-2 has been suggested (Grifoni et al., 2020), the key step in the immune response is undoubtedly its recognition by naïve, and not memory, T cells. Early and efficient virus-specific CD8+ T cell responses and CD4+ T cell-dependent antibody responses of sufficient magnitude against SARS-CoV-2 would probably be protective (Zhao J. et al., 2016). However, age-related alterations in the T cell compartment could lead to a failure to develop protective immunity in old patients. As previously mentioned, thymic involution is the main driver of the reduced absolute numbers and percentages of peripheral naïve T cells observed in old individuals. Their homeostatic peripheral proliferation is thought to compensate for this loss but can lead to the outgrowth of certain T cell clones at the expense of others, with shrinkage of the T cell repertoire. As a consequence, the pool of naïve T cells decreases with age with quantitatively and qualitatively impairs de novo CD8+ T-cell responses (Briceño et al., 2016). In parallel, there is an expansion of the memory T cell pool, probably due to chronic or persistent infections, most commonly with Cytomegalovirus, which cause specific T cells to clonally expand through repetitive stimulation. One of the phenotypic characteristics of these persistent stimulated T cells is the progressive downregulation of the costimulatory receptor CD28 that is definitively lost in terminally differentiated T cells (Pangrazzi et al., 2020). These CD28– senescent T cells are characterized by low proliferative capacity, shortening of telomeres and express senescent markers, such as programmed cell death protein 1 (PD-1). These age-associated changes in T cell subset distribution, especially in CTL, critically affect primary immune responses against viruses (Pawelec, 2018; Aiello et al., 2019). A recent study performed by our group has highlighted differences in the lymphocyte subset distribution in both helper and cytotoxic compartments between females and males during aging (Ligotti et al., 2021). These immune gender differences during aging and in case fatality rates in COVID-19 disease support the need to incorporate investigation of biological factors underlying differences in immune responses to SARS-CoV-2 between females and males in order to identify targeted therapeutic interventions aimed to improve antiviral immune function (Bunders and Altfeld, 2020).

Despite differences in patient stratification, several studies have reported lymphopenia, affecting all lymphocyte subsets, in a significant proportion of patients with severe diseases, both males and females, such that it can be considered, together with hyper-cytokinaemia, a signature for severe COVID-19 infection and pneumonia (Bermejo-Martin et al., 2020). The occurrence of lymphopenia (lymphocyte count < 0.8–1.1 × 109/L, depending on the cut-off value used) was reported in 85% (Yang et al., 2020), 72.3% (Liu K. et al., 2020; Zhan et al., 2020), 66.7% (Meng Y. et al., 2020), 63% (Huang et al., 2020), and 42.9% (Chen G. et al., 2020) of infected cases, with the highest percentages observed in critically ill patients. Specifically, significant decreases in absolute T cell counts, TH cells, and CTL cells were observed in the COVID-19 patients compared to healthy controls, but accentuated in the severe cases (Cui et al., 2020; Wang F. et al., 2020), indicating SARS-CoV-2 infection has a negative impact on T-cell mediated immunity. In older COVID-19 patients (median age 71 years), low lymphocytes count was a strong predictor of poor outcome while high lymphocyte levels were predictive of better outcome (Wang L. et al., 2020). This peripheral lymphopenia could reflect the recruitment of lymphocytes from the blood to the infected site in response to combinations of different chemokines expressed by airway and alveolar blood vessel endothelial cells (Alon et al., 2021). A meta-analysis of the mean difference in lymphocyte counts at admission between patients with good and with poor COVID-19 outcomes has shown that lymphopenia was significantly associated with severe COVID-19 and this association was affected by age but not by sex (Zhao Q. et al., 2020). However, some studies have suggested that the male sex is inversely associated with lymphocyte count, especially in patients with comorbidity (Meng Y. et al., 2020; Qin et al., 2020), although not always confirmed (Zhao G. et al., 2021). In the peripheral blood of SARS-CoV-2-infected patients, both CD4+ and CD8+ T cell blood counts are dramatically decreased compared to healthy controls, with the highest evidence observed in severe cases (de Candia et al., 2021). Nevertheless, male COVID-19 patients have shown lower CD4+ T cell and higher CD8+ T cell proportions than female patients, indicating a possible more severe immune dysregulation (Zhao G. et al., 2021; Table 4).

**TABLE 4:** Observed fluctuation in T cell subsets in adult COVID-19 patients.

T cell subsets	Observation	Analyzed patients	Compared group(s)	References
Total CD3+CD19– T lymphocyte	Reduction (%)	Deceased	Survivors	Cui et al., 2020
	Reduction (#)	Deceased	Survivors	Cui et al., 2020; Belaid et al., 2021
	Reduction (%)	Cases	Recovered	Mathew et al., 2020
	Reduction (%)	Cases	HD	Mathew et al., 2020
	Reduction (#)	Severe^η^	Mild^ζ^ /moderate^θ^	Song et al., 2020; Belaid et al., 2021
	No change (%)	Severe^η^	Mild^ζ^	Song et al., 2020
CD3+CD4+ Helper T lymphocyte	Reduction (%)	Deceased	Survivors	Cui et al., 2020
	Reduction (#)	Deceased	Survivors	Cui et al., 2020
	Reduction (#)	Pneumonia patients	HD	Wang F. et al., 2020
	Reduction (#)	Severe^η^	Mild^ζ^ /moderate^θ^	Song et al., 2020; Wang F. et al., 2020; Belaid et al., 2021; Taghiloo et al., 2021
	No change (%)	Severe^η^	HD	De Biasi et al., 2020
	Reduction (#)	Severe^η^	HD	De Biasi et al., 2020
	Reduction (%)	Cases	Recovered	Mathew et al., 2020
	Reduction (%)	Cases	HD	Mathew et al., 2020
	No change (%)	Severe^η^	Mild^ζ^	Song et al., 2020
CD3+CD8+ Cytotoxic T lymphocyte	Reduction (%)	Deceased	Survivors	Cui et al., 2020
	Reduction (#)	Deceased	Survivors	Cui et al., 2020; Belaid et al., 2021
	Reduction (#)	Pneumonia patients	HD	Wang F. et al., 2020
	Reduction (#)	Severe^η^	Mild^ζ^ /moderate^θ^	Song et al., 2020; Wang F. et al., 2020; Belaid et al., 2021; Taghiloo et al., 2021
	No change (%)	Severe^η^	HD	De Biasi et al., 2020
	Reduction (#)	Severe^η^	HD	De Biasi et al., 2020
	Reduction (%)	Cases	Recovered	Mathew et al., 2020
	Reduction (%)	Cases	HD	Mathew et al., 2020
	No change (%)	Severe^η^	Mild	Song et al., 2020
CCR7+CD45RA+CD28+CD27+ CD4+ naïve	No change (%)	Severe^η^	HD	De Biasi et al., 2020
	Reduction (#)	Severe^η^	HD	De Biasi et al., 2020
CCR7-CD45RA+CD28+CD27+/- CD4+ central memory	No change (%)	Severe^η^	HD	De Biasi et al., 2020
	Reduction (#)	Severe^η^	HD	De Biasi et al., 2020
	No change (%)	Severe^η^	HD	De Biasi et al., 2020
	Reduction (#)	Severe^η^	HD	De Biasi et al., 2020
CCR7-CD45RA+CD28-CD27+/- CD4+ terminal effector	Increase (%)	Severe^η^	HD	De Biasi et al., 2020
	No change (#)	Severe^η^	HD	De Biasi et al., 2020
CCR7-CD45RA+CD27- CD4+ naïve terminal effector	Increase (%)	Cases	Recovered	Mathew et al., 2020
	Increase (%)	Cases	HD	Mathew et al., 2020
CD38+HLA-DR+ CD4+ activated	No change (#)	Severe^η^	HD	De Biasi et al., 2020
	Increase (%)	Severe^η^	HD	De Biasi et al., 2020
	Increase (%)	Mild^ζ^	HD	Song et al., 2020
	Increase (%)	Severe^η^	HD	Song et al., 2020
	Increase (%)	Deceased	Survivors	Belaid et al., 2021
	Increase (%)	Cases	Recovered	Mathew et al., 2020
	Increase (%)	Cases	HD	Mathew et al., 2020
	No change (%)	Severe^η^	Mild^ζ^ /moderate^θ^	Song et al., 2020; Belaid et al., 2021
PD-1+CD57+ CD4+ exhausted/senescent	Increase (%)	Severe^η^	HD	De Biasi et al., 2020
	No change (#)	Severe^η^	HD	De Biasi et al., 2020
CCR7+CD45RA+CD28+CD27+ CD8+ naïve	Reduction (%)	Severe^η^	HD	De Biasi et al., 2020
	Reduction (#)	Severe^η^	HD	De Biasi et al., 2020
CCR7+CD45RA+ CD8+ naïve	Reduction (%)	Severe^η^	Mild^ζ^ /moderate^θ^	Belaid et al., 2021
	Reduction (%)	Deceased	Survivors	Belaid et al., 2021
CCR7-CD45RA+CD28+CD27+/- CD8+ central memory	Reduction (%)	Severe^η^	HD	De Biasi et al., 2020
	Reduction (#)	Severe^η^	HD	De Biasi et al., 2020
CCR7-CD45RA-CD28+/-CD27+/- CD8+ effector memory	No change (%)	Severe^η^	HD	De Biasi et al., 2020
	No change (#)	Severe^η^	HD	De Biasi et al., 2020
CCR7-CD45RA-CD8+ effector memory	Increase (%)	Deceased	Survivors	Belaid et al., 2021
CCR7-CD45RA+CD28-CD27+/- CD8+ terminal effector	Increase (%)	Severe^η^	HD	De Biasi et al., 2020
	No change (#)	Severe^η^	HD	De Biasi et al., 2020
CCR7-CD45RA+CD27- CD8+ naïve terminal effector	Increase (%)	Cases	Recovered	Mathew et al., 2020
	Increase (%)	Cases	HD	Mathew et al., 2020
CD38+HLA-DR+ CD8+ activated	Increase (%)	Severe^η^	HD	De Biasi et al., 2020
	Increase (#)	Severe^η^	HD	De Biasi et al., 2020
	Increase (%)	Mild^ζ^	HD	Song et al., 2020
	Increase (%)	Severe^η^	HD	Song et al., 2020
	Increase (%)	Deceased	Survivors	Belaid et al., 2021
	Increase (%)	Severe^η^	Mild^ζ^	Song et al., 2020
	Increase (%)	Cases	Recovered	Mathew et al., 2020
	Increase (%)	Cases	HD	Mathew et al., 2020
	No change (%)	Severe^η^	Mild^ζ^ /moderate^θ^	Belaid et al., 2021
PD-1+CD57+ CD8+ exhausted/senescent	Increase (%)	Severe^η^	HD	De Biasi et al., 2020
	No change (#)	Severe^η^	HD	De Biasi et al., 2020
BAL T cells	Reduction (%)	Severe^η^ /Critical^ι^	Moderate^θ^	Liao et al., 2020
	Increase (%)	Moderate^θ^	HD	Liao et al., 2020

*T cell subset, peripheral blood T cell subset analyzed in the reported studies; (%), as frequency values; (#), as absolute number; Cases, COVID-19 patients (irrespective of severity); HD, healthy donors; Recovered, non-hospitalized subjects who had recovered from SARS-CoV-2 infection.*

*^ζ^Patients with or without pneumonia admitted to general wards not requiring intensive care.*

*^η^Patients experiencing the following: respiratory failure, respiratory rate > 30 bpm, oxygen saturation < 93% at rest, arterial partial pressure of oxygen (PaO_2_)/fraction of inspired oxygen (FiO_2_) (PaO_2_/FiO_2_) ratio < 300 mmHg.*

*^θ^Fever, respiratory symptoms, and pneumonia evidenced by computed tomography.*

*^ι^Any of the following: requirement for mechanical ventilation, shock, or concomitant organ failure.*

Analysis of T cells in BAL of COVID-19 patients has identified lower CD8+ T cell levels in patients with severe infection than in patients with moderate infection, apparently against the hypothesis of sequestration on the site of infection. On the other hand, CD8+ T cells in BAL from patients with moderate infection have shown upregulation of genes involved in differentiation, activation, migration and cytokine-related pathways compared with severe cases (Liao et al., 2020; Table 4).

Despite the absolute counts of CD8+ T cells decreased, analysis of T cell activation has revealed a significant increment in the frequency and absolute numbers of activated (co-expressing HLA-DR and CD38) CD8+ T cells and increased multiple cytotoxic granules expression in COVID-19 pneumonia patients (De Biasi et al., 2020; Song et al., 2020; Chen Q. et al., 2021). Analysis of post-mortem lung tissue from 16 patients with COVID-19 has correlated the presence of abundant infiltrating activated CD8+ T cells (CD38, GZMA, GZMB, CCR5) with a specific immunopathological pattern characterized by low local expression of interferon stimulated genes, low viral counts, massive lung damage, and late death (Nienhold et al., 2020). Based on these pieces of evidence, it has been suggested that this aberrant activation of CTLs in patients with severe COVID-19 disease could play an important role in the pathogenesis of SARS-CoV-2 infection (Song et al., 2020). However, CD8+ T cells from older COVID-19 patients with mild disease do not show increased production of perforin and granzymes, probably due to high background levels of cytotoxic molecules (Westmeier et al., 2020; Table 4).

A significant increment in the frequency, but not in the absolute count, of activated CD4+ T cells or exhausted/senescent (PD-1+CD57+) peripheral CD4+ and CD8+ T cells was also observed (De Biasi et al., 2020). When gender differences were considered, this increase in percentages of activated and terminally differentiated T cells being significant in female but not in male patients compared with healthy controls, suggesting a more robust T cell response among female patients. Although these differences were observed in both CD4+ and CD8+ T cells, more significant differences were found in the cytotoxic subset (Chen and John Wherry, 2020; Takahashi et al., 2020; Table 4). The implications and mechanisms underlying increased CD8+ activity are unknown in the context of this novel virus but may play a role in driving the immunopathogenesis of COVID-19.

The characterization of the T cell subsets in a cohort of 39 COVID-19 with a median age of 64 years (range 35–94) has revealed that a decrease in both the percentage and the absolute number of naïve CD45RA+CCR7+ CD8+ T cells and in the absolute number of naïve CD45RA+CCR7+ CD4+ T cells (De Biasi et al., 2020; Belaid et al., 2021). A similar trend was observed as CCR7+CD45RA- central memory T cells, while the percentage, but not the absolute number of terminal effector CCR7-CD45RA+ T cells was higher, although with a decreased proliferation index suggesting a lack of clonal expansion after activation (De Biasi et al., 2020). Compared to hospitalized COVID-19 patients during acute disease, patients at 3–6 months of convalescence showed higher proportions of CD4+ and CD8+ CCR7–CD45RA+ effector T cells and reduced expression of the proliferation marker Ki-67. In male patients, disease progression was associated with higher age and lower CD8+ T cell response (IFN-γ production and activation), while these correlations were not seen in females. On the contrary, female patients showed greater T cell response, characterized by higher percentages of activated and terminally differentiated CD8+ T cells (Takahashi et al., 2020; Table 4).

CD4+ and CD8+ T cells in acute COVID-19 patients showed a substantial reduction of CXCR3 and CXCR5 expression, irrespective of disease severity, that is recovered upon convalescence (Shuwa et al., 2021). In another study, when compared to their younger counterparts and to age-matched healthy donors, over 80-year-olds COVID-19 patients showed a more pronounced reduction in CD8+ T cell count. Moreover, percentages of naïve CD45RO- CCR7+ CD28+ CD8+ T cells were markedly reduced in 29- to 79-year-old group of COVID-19 patients, suggesting an ongoing cytotoxic response during SARS-Cov-2 infection, but these differences were not evident in the over 80 group, probably due to the reduced pool of naïve T cells in the old individuals. Likewise, the increase in CD8+ CD45RO- CCR7- CD28- terminally differentiated effector and CD45RO+ CCR7- CD28- effector memory T cells was more evident in the younger group than in the 80- to 96-year-old age group (Westmeier et al., 2020). CD4+ memory responses to SARS-CoV-2 were detected in 100% of recovered patients, while 70% established CD8+ memory responses. Most of T cell reactivity to the viral Spike protein was dependent on CD4+ T cells and these responses were correlated with anti-SARS-Cov-2 IgG and IgA titres in COVID-19 cases (Grifoni et al., 2020). A sophisticated and detailed analysis of T cell immunophenotype in COVID-19 patients has revealed the heterogeneity of the immune response to SARS-CoV-2 infection ranging from robust CD8+ and/or CD4+ T cell activation and proliferation to minimal detectable responses compared to healthy donors (Mathew et al., 2020). This suggests that the relationship between acquired immunity and COVID-19 pathogenesis is complex and must be taken into account in the design of therapies and vaccines (Table 4).

Most analyses were not stratified by gender. It therefore remains to be established whether cell-mediated immune response may differ between the two sexes and whether this affects the durability of immunity and vaccine approaches. Moreover, given the multitude of well-documented age-related changes in the composition and responsiveness of the acquired immune system, further comparative studies between young and old are needed to investigate differences in virus-specific T cells responses. Indeed, any intervention aimed at improving immune status in old individuals should be targeted and individualized, especially in a context such as COVID-19 that is fatal to a large proportion of this population.

## Acquired Immunity: B Lymphocytes

B cells function in the humoral immunity component of the acquired immune system, producing antibodies that are not secreted, but inserted into the plasma membrane where they serve as a part of B-cell receptors. When a naïve or memory B cell is activated by an antigen, it proliferates and may differentiate into a short living plasmablast, or a long living plasma cell (both known as antibody-secreting cells – ASCs) – it is not known if plasma cells represent a further maturation step of early plasmablasts (Jourdan et al., 2009; Khodadadi et al., 2019).

Immunosenescence impacts B lymphocytes at multiple levels. During aging, B cells show a decline in the number of newly produced clones flanked by a contraction of their repertoire, producing a variable level of accumulation of memory cells and an increase in the appearance of signs of exhaustion (Giefing-Kröll et al., 2015; Aiello et al., 2019; Crooke et al., 2019; Ma et al., 2019; Lian et al., 2020). The age associated decline in the amount of B cells appears more dramatic in men than in women, and menopausal related changes in B cell numbers are counteracted by hormone replacement therapy (Giefing-Kröll et al., 2015; Márquez et al., 2020a). Also, during aging women exhibit more pronounced chromatin accessibility at B cell *loci* than older male individuals, who suffer a downregulation of B cell-specific genes (Márquez et al., 2020a).

Changes in B cell distribution during COVID-19 affect the quality and intensity of SARS-CoV-2 elicited immune response, accounting for infection resolution and persistence of symptoms after viral clearance (Huang et al., 2021; Long et al., 2021; Shuwa et al., 2021; Wheatley et al., 2021). This was demonstrated also from a functional point of view, with both somatic hypermutation in B cell receptor (BCR) and higher antibody titres correlating with disease severity (Schultheiß et al., 2020; Okba et al., 2020). Thus, COVID-19 related B lymphocyte alterations determine not only disease severity but also the entity of long-term consequences and may have potential prognostic applications (Detsika et al., 2021; Kim C.W. et al., 2021; Kos et al., 2021; Shuwa et al., 2021).

### Total B Cells

During aging a contraction of both number and percentage of peripheral B cells was described multiple times, with a more visible reduction in older males (Bulati et al., 2014; Giefing-Kröll et al., 2015; Hazeldine and Lord, 2020; Lian et al., 2020; Márquez et al., 2020b,a; Vellas et al., 2020). In COVID-19 the analysis of B cell changes according to a gender-based stratification of patients revealed that only the frequency of B cells was higher in both non-ICU male and female patients than in controls, reaching a statistically significant result narrowly when the comparison was performed between the two female groups (Takahashi et al., 2020). However, no sex or age correlated differences in B cell counts of COVID-19 patients were documented (Jurado et al., 2020; Takahashi et al., 2020; Jin et al., 2021).

In gender pooled reports about the total number and percentage of B cells in COVID-19+ subjects, data vary from a study to another on the basis of both the choice of the confronted parameter (number of cells rather than percentage) and the adopted criteria to stratify the analyzed subjects according to severity (Carsetti et al., 2020; Sosa-Hernández et al., 2020; Detsika et al., 2021; Kos et al., 2021; Shuwa et al., 2021; Xiong et al., 2021). Numerous papers are available documenting the invariability, the decrease and the increase of B cell numbers and frequency in various categories of COVID-19 patients (Table 5). This lack of homogeneity mirrors the absence of an agreement about the stratification of COVID-19 cases according to severity. In addition, the selected statistical analysis strategy, especially in the presence of multiple comparisons, may “mask” some statistically significant results, requiring a larger sample size or a different statistical approach to reveal relevant differences among the compared categories (Jurado et al., 2020; Sosa-Hernández et al., 2020; Woodruff et al., 2020; d’Alessandro et al., 2021; Newell et al., 2021; Shuwa et al., 2021). As a result, the abundance of confounding reports slows down the extrapolation of prognostic and predictive pieces of information from published reports.

**TABLE 5:** Observed fluctuation in B cells in adult COVID-19 patients.

B cells	Observation	Analyzed patients	Compared group(s)	References
Total peripheral blood B cells	No change (#)	Intubated	Non-intubated	Detsika et al., 2021
	No change (#)	Intubated (admission)	Intubated (recovery)	Detsika et al., 2021
	No change (#)	Severe^+^ (admission)	Mild* (admission)	Jurado et al., 2020
	No change (#)	Moderate^&^ (admission)	Mild* (admission)	Jurado et al., 2020
	No change (#)	Severe^+^ (admission)	Moderate^&^ (admission)	Jurado et al., 2020
	No change (#)	Critical^§^	Severe^Ł^	Liu Y. et al., 2021
	No change (#)	Cases	HD	d’Alessandro et al., 2021
	No change (#)	Severe^¿^	Non-severe^¡^	d’Alessandro et al., 2021
	Reduction (#)	Deceased	Survivors	Cantenys-Molina et al., 2021; Kos et al., 2021; Xiong et al., 2021
	Reduction (#)	Critical^§^	Moderate^@^	Liu Y. et al., 2021
	Reduction (#)	Severe°	Non-severe∧	Zhang W. et al., 2020
	Reduction (#)	Composite^α^	Non-composite^β^	Zhang W. et al., 2020
	Increase (#)	ICU	NCU	Kos et al., 2021
	Increase (#)	ICU	NCU (non-COVID-19)	Kos et al., 2021
	Increase (#)	ICU	ICU (non-COVID-19)	Kos et al., 2021
	No change (%)	Convalescent	HD	Newell et al., 2021
	No change (%)	Severe^¿^	Non-severe^¡^	d’Alessandro et al., 2021
	No change (%)	Severe^+^ (admission)	Mild* (admission)	Jurado et al., 2020
	No change (%)	Severe^+^ (discharge)	Mild* (discharge)	Jurado et al., 2020
	No change (%)	Moderate^&^ (admission)	Mild* (admission)	Jurado et al., 2020
	No change (%)	Moderate^&^ (discharge)	Mild* (discharge)	Jurado et al., 2020
	No change (%)	Severe^+^ (admission)	Moderate^&^ (admission)	Jurado et al., 2020
	No change (%)	Severe^+^(discharge)	Moderate^&^(discharge)	Jurado et al., 2020
	Reduction (%)	ICU	NCU	Kos et al., 2021
	Reduction (%)	ICU	NCU (non-COVID-19)	Kos et al., 2021
	Reduction (%)	ICU	ICU (non-COVID-19)	Kos et al., 2021
	Reduction (%)	Severe^γ^	Mild^δ^	Shuwa et al., 2021
	Reduction (%)	Severe^γ^	Moderate^ε^	Shuwa et al., 2021
	Reduction (%)	Severe^ζ^	Asymptomatic	Carsetti et al., 2020
	Increase (%)	Cases	HD	Woodruff et al., 2020
	Increase (%)	Severe^η^	Mild/moderate^θ^	Sosa-Hernández et al., 2020
	Increase (%)	Cases	Convalescent	Files et al., 2021
Bone marrow and spleen total B cells	Reduction (#)	Deceased	Controls^$^	Ihlow et al., 2021
BAL B cells	Reduction (%)	Severe^/^	Mild^/^	Kim C.W. et al., 2021
	Increase (%)	Mild^/^	HD^/^	Kim C.W. et al., 2021
	Increase (#)	Severe^/^	Mild^/^	Kim C.W. et al., 2021
	Increase (#)	Severe^/^	HD^/^	Kim C.W. et al., 2021

BAL, bronchoalveolar lavage; (%), as frequency values; (#), as absolute number; Cases, COVID-19 patients (irrespective of severity); ICU, intensive care unit patients; HD, healthy donors; NCU, normal care unit patients; NCU (non-COVID-19), normal care unit patients treated for other reasons than COVID-19; ICU (non-COVID-19), intensive care unit patients treated for other reasons than COVID-19; asymptomatic, patients who tested positive but had no symptoms. *Clinical mild symptoms with no abnormal radiological findings.

*^+^Non-severe pneumonia.*

*^&^As defined by the physician in charge or meeting at least one of the following criteria: acute respiratory distress, shock, admission to the intensive care unit -ICU-; patients with a fatal outcome were also included in this group.*

*^§^With any of the following: (1) respiratory failure requiring mechanical ventilation; (2) shock; or (3) other organ failure requiring monitoring and treatment in ICU.*

*^Ł^Individuals experiencing any of the following: (1) respiratory distress, respiratory rate ≥ 30 breaths/min; (2) oxygen saturation on room air ≤ 93% at rest; (3) oxygenation index PaO_2_/FIO_2_ ≤ 300 mmHg; or (4) lung infiltrates > 50% within 24–48 h.*

*^$^Age matched subjects who died for cardiac conditions.*

*^¿^ICU admission, mechanical ventilation, or high-flow oxygen therapy.*

*^¡^Patients who were not experiencing ICU admission, mechanical ventilation, or high-flow oxygen therapy.*

*^@^Fever and other respiratory tract symptoms with pneumonia on chest computed tomography. °Respiratory rate ≥ 30/min, a pulse oxygen saturation ≤ 93% on room air, oxygenation index ≤ 300 mmHg, respiratory failure for which invasive ventilation was necessary, signs of shock (circulatory failure), organ failure and ICU care. ∧Mild symptoms + normal radiology findings in both lungs, and fever, cough, and other respiratory symptoms + radiological signs of pneumonia.*

*^α^Patients experiencing admission to the ICU, mechanical ventilation, or death.*

*^β^Patients who did not experience admission to the ICU, mechanical ventilation, or death.*

*^γ^Patients with > 10L or 60% supplemental oxygen, managed in ICU.*

*^δ^Patients with < 3L or 28% supplemental oxygen.*

*^ε^Patients with < 10L or <60% supplemental oxygen, requiring non-invasive ventilation (NIV) or continuous positive airway pressure (CPAP).*

*^ζ^Patients with clinical signs of pneumonia with one of the following: respiratory rate > 30 breaths/min, severe respiratory distress, or SpO_2_ < 90% on room air.*

*^η^Patients experiencing the following: respiratory failure, respiratory rate > 30 bpm, oxygen saturation < 92% at rest, arterial partial pressure of oxygen (PaO_2_)/fraction of inspired oxygen (FiO_2_) (PaO_2_/FiO_2_) ratio < 300 mmHg.*

*^θ^Fever, signs of airway disease, with or without a tomographic image indicating pneumonia.*

*^/^No stratification criteria neither statistically significance data provided.*

Despite these contradictory data, a recent metanalysis demonstrated a statistically significant decrease in B cells in severe disease cases vs. non severe disease group (Akbari et al., 2020).

### Transitional and Naïve B Cells

Immunosenescence is associated with a decrease in CD27-IgD+ naïve B lymphocytes and the consequential reduction in B cell repertoire diversity (Cancro et al., 2009; Crooke et al., 2019; Ma et al., 2019; Oh et al., 2019). To the best of our knowledge, no naïve B cell specific molecular and numerical analysis was performed as a function of age and gender in COVID-19, and naïve B cells are reported as mostly unchanged in their frequency irrespective of disease severity (Table 6; Sosa-Hernández et al., 2020; Long et al., 2021; Shuwa et al., 2021). However, frequency of CD19+CD27loIgM+IgD+ mature naïve B cells seemed to be correlated with seroconversion, considering that percentages recorded for this B subset were higher in patients with high seroconversion index values vs. subjects with low seroconversion index values (seroconversion indices were estimated by summing the *Z*-scores for each of the four seroconversion assays used by the authors – see Galbraith et al., 2021 for details) (Galbraith et al., 2021).

**TABLE 6:** Observed fluctuation in naïve and transitional B cells in adult COVID-19 patients.

B cell subset	Observation	Analyzed patients	Compared group(s)	References
CD27-IgD+ naïve	No change (%)	Severe^γ^	Moderate^ε^	Shuwa et al., 2021
	No change (%)	Severe^γ^	Mild^δ^	Shuwa et al., 2021
	No change (%)	Severe^γ^	HD	Shuwa et al., 2021
	No change (%)	Moderate^ε^	Mild^δ^	Shuwa et al., 2021
	No change (%)	Moderate^ε^	HD	Shuwa et al., 2021
	No change (%)	Mild^δ^	HD	Shuwa et al., 2021
	No change (%)	Cases	Recovered	Mathew et al., 2020
	No change (%)	Cases	HD	Mathew et al., 2020
	No change (%)	Recovered	HD	Mathew et al., 2020
	Increase (%)	Severe^κ^	Prepandemic controls	Acosta-Ampudia et al., 2021
IgD+CD38–/+ naïve	No change (%)	Severe^η^	Critical^ι^	Sosa-Hernández et al., 2020
	No change (%)	Severe^η^	Mild/moderate^θ^	Sosa-Hernández et al., 2020
	No change (%)	Severe^η^	HD	Sosa-Hernández et al., 2020
	No change (%)	Critical^ι^	Mild/moderate^θ^	Sosa-Hernández et al., 2020
	No change (%)	Critical^ι^	HD	Sosa-Hernández et al., 2020
	No change (%)	Mild/moderate^θ^	HD	Sosa-Hernández et al., 2020
CD21+CD27– naïve	No change (%)	Recovered	HD	Long et al., 2021
Naïve	Increase (%)	Mild^/^	HD	Huang et al., 2021
	Increase (%)	Moderate^/^	HD	Huang et al., 2021
	Increase (%)	Cured^/^	HD	Huang et al., 2021
CD24hiCD38hi transitional	Reduction (%)	Severe^γ^	HD	Shuwa et al., 2021
	Reduction (%)	Severe^γ^	Convalescent	Shuwa et al., 2021
CD24+CD38hi transitional	No change (%)	Severe^η^	HD	Sosa-Hernández et al., 2020
	Reduction (%)	Severe^η^	Mild/moderate^θ^	Sosa-Hernández et al., 2020
	Reduction (%)	Critical^ι^	Mild/moderate^θ^	Sosa-Hernández et al., 2020
	Increase (%)	Mild/moderate^θ^	HD	Sosa-Hernández et al., 2020
CD27-CD38int CD24+ transitional	Reduction (%)	ICU	Outpatients	Woodruff et al., 2020

*B cell subset, peripheral blood B cell subset analyzed in the reported studies; (%), as frequency values; (#), as absolute number; Cases, COVID-19 patients (irrespective of severity); ICU, intensive care unit patients; Recovered, non-hospitalized subjects who had recovered from SARS-CoV-2 infection; HD, healthy donors; Outpatients, outpatients with milder disease.*

*^γ^Patients with > 10 L or 60% supplemental oxygen, managed in ICU.*

*^δ^Patients with < 3L or 28% supplemental oxygen.*

*^ε^Patients with < 10L or <60% supplemental oxygen, requiring non-invasive ventilation (NIV) or continuous positive airway pressure (CPAP).*

*^ζ^Patients with clinical signs of pneumonia with one of the following: respiratory rate > 30 breaths/min, severe respiratory distress, or SpO_2_ < 90% on room air.*

*^η^Patients experiencing the following: respiratory failure, respiratory rate > 30 bpm, oxygen saturation < 92% at rest, arterial partial pressure of oxygen (PaO_2_)/fraction of inspired oxygen (FiO_2_) (PaO_2_/FiO_2_) ratio < 300 mmHg.*

*^θ^Fever, signs of airway disease, with or without a tomographic image indicating pneumonia.*

*^ι^Any of the following: requirement for mechanical ventilation, shock, or concomitant organ failure.*

*^κ^Respiratory distress, i.e., ≥30 breaths/min. in resting state, oxygen saturation of 90% or less on room air; or arterial partial pressure of oxygen (PaO_2_)/fraction of inspired oxygen (FiO_2_) of 300 or less. ^/^No stratification criteria provided.*

On the contrary, transitional B cells tend to remain stable in adults (Perez-Andres et al., 2010; Blanco et al., 2018) and may exert immunomodulatory functions producing IL-10 (Zhou Y. et al., 2020). In COVID-19 literature, data about transitional B cell frequency according to disease severity are not fully homogeneous, despite the majority of data suggest that a reduction in transitional B cells may contribute to more severe disease (Table 6; Sosa-Hernández et al., 2020; Woodruff et al., 2020; Shuwa et al., 2021).

Some of the lack of reproducibility (leading to the same methodological considerations) that we emphasized in the previous paragraph may be also encountered in those studies dissecting alterations of transitional and naïve B cells in COVID-19 patients. In addition, a shared agreement about the terms by which expressing the immunophenotypic features of the described subset would be extremely beneficial to ensure the comparability of data (Table 6).

### Memory B Cells

Dysregulation of B cell production, survival and turnover in older subjects affects the ability to respond to new antigens (thus influencing response to vaccination) and may cause the accumulation of class switched memory cells (IgD-CD27+), although the eventuality of such an effect is still a matter of debate (Ventura et al., 2017; Aiello et al., 2019; Crooke et al., 2019; Hazeldine and Lord, 2020). Also, age-associated B cells (ABCs), defined as CD11b+CD11c+CD21low/-T-bet+, increase with age, and accounts for a reduction in the generation of new B cells, the release of pro-inflammatory cytokines and autoantibody production in autoimmune diseases (Rubtsova et al., 2015; Hagen and Derudder, 2020; Pietrobon et al., 2020; Sachinidis et al., 2020; Kim C.W. et al., 2021).

As regards B cell homeostasis in COVID19+ patients, to the best of our knowledge only one age focused study explored the effect of immunosenescence on memory B cell dynamics, demonstrating that percentage of CD21+CD27+ memory B cells exhibited a direct correlation with patients age (Kuri-Cervantes et al., 2020). Data from both COVID-19 patients (Lenti et al., 2020; Ogega et al., 2021; Shuwa et al., 2021) and recovered COVID-19 subjects (Long et al., 2021) showed no effects in terms of memory B cell accumulation, resembling those reports documenting a similar scenario during aging (Crooke et al., 2019). These findings were not confirmed by all the available studies, mainly because of differences in the patient stratification criteria, insufficient sample size and inconsistency in the evaluated immunophenotypic features that were used to detect the desired subset (Table 7; Carsetti et al., 2020; Lenti et al., 2020; Mathew et al., 2020; Sosa-Hernández et al., 2020; Acosta-Ampudia et al., 2021; Shuwa et al., 2021).

**TABLE 7:** Observed fluctuation in B cells in adult COVID-19 patients.

B cells	Observation	Analyzed patients	Compared group(s)	References
CD21+CD27+ memory	Reduction (%)	Severe^ρ^	HD	Kuri-Cervantes et al., 2020
CD27+IgD+ unswitched memory	No change (%)	Severe^γ^	Moderate^ε^	Shuwa et al., 2021
	No change (%)	Severe^γ^	Mild^δ^	Shuwa et al., 2021
	No change (%)	Severe^γ^	HD	Shuwa et al., 2021
	No change (%)	Moderate^ε^	Mild^δ^	Shuwa et al., 2021
	No change (%)	Moderate^ε^	HD	Shuwa et al., 2021
	No change (%)	Mild^δ^	HD	Shuwa et al., 2021
	Increase (%)	Severe^κ^	Prepandemic controls	Acosta-Ampudia et al., 2021
	Reduction (%)	Severe^η^	HD	Sosa-Hernández et al., 2020
	Reduction (%)	Critical^ι^	HD	Sosa-Hernández et al., 2020
	Reduction (%)	Cases	Recovered	Mathew et al., 2020
	Reduction (%)	Cases	HD	Mathew et al., 2020
IgD+IgM+ unswitched memory	No change (%)	Severe	Mild	Ogega et al., 2021
	No change (%)	Severe	HD	Ogega et al., 2021
	No change (%)	Mild	HD	Ogega et al., 2021
CD27+IgD- switched memory	No change (%)	Severe^γ^	Moderate^ε^	Shuwa et al., 2021
	No change (%)	Severe^γ^	Mild ^δ^	Shuwa et al., 2021
	No change (%)	Severe^γ^	HD	Shuwa et al., 2021
	No change (%)	Moderate^ε^	Mild ^δ^	Shuwa et al., 2021
	No change (%)	Moderate^ε^	HD	Shuwa et al., 2021
	No change (%)	Mild^δ^	HD	Shuwa et al., 2021
	Reduction (%)	Severe^η^	HD	Sosa-Hernández et al., 2020
	Reduction (%)	Cases	Recovered	Mathew et al., 2020
	Reduction (%)	Cases	HD	Mathew et al., 2020
CD27-IgM- switched memory	Reduction (%)	Severe^ζ^	Mild^π^	Carsetti et al., 2020;
CD38+/–IgM–IgD– switched memory	No change (%)	Severe^λ^	Mild^μ^	Ogega et al., 2021
	No change (%)	Severe^λ^	HD	Ogega et al., 2021
	No change (%)	Mild^μ^	HD	Ogega et al., 2021
	No change (#)	Cases	HD	Lenti et al., 2020
	No change (#)	Cases	Hypofunction^ν^	Lenti et al., 2020
	No change (#)	Cases	Splenectomy^ξ^	Lenti et al., 2020
CD27+IgD-IgM-CD38+ switched memory	Increase (%)	Severe^κ^	Prepandemic controls	Acosta-Ampudia et al., 2021
IgM+ memory	Reduction (%)	Severe^ζ^	Asymptomatic	Carsetti et al., 2020
	Reduction (%)	Severe^ζ^	Mild^π^	Carsetti et al., 2020
	Reduction (%)	Cases	HD	Lenti et al., 2020
IgM-IgG- memory	Increase (%)	Severe^ζ^	Asymptomatic	Carsetti et al., 2020
	Increase (%)	Severe^ζ^	Mild^π^	Carsetti et al., 2020
CD27-IgD- double negative memory	Increase (%)	Severe^γ^	HD	Shuwa et al., 2021;
	Increase (%)	Convalescent	HD	Shuwa et al., 2021;
	Increase (%)	Cases	Recovered	Mathew et al., 2020
	Increase (%)	Cases	HD	Mathew et al., 2020
CD38–/+CD24–CD21+CD11c– DN1	Reduction (%)	Severe^η^	Mild/moderate^θ^	Sosa-Hernández et al., 2020
	Reduction (%)	Critical ^ι^	Mild/moderate ^θ^	Sosa-Hernández et al., 2020
CD38–CD24-CD21–CD11c+ DN2	Increase (%)	Severe^η^	Mild/moderate^θ^	Sosa-Hernández et al., 2020
	Increase (%)	Severe^η^	Critical^ι^	Sosa-Hernández et al., 2020
CD38–/+CD24–CD21–CD11c– DN3	Increase (%)	Severe^η^	HD	Sosa-Hernández et al., 2020
	Increase (%)	Critical^ι^	Mild/moderate^θ^	Sosa-Hernández et al., 2020
	Increase (%)	Critical^ι^	HD	Sosa-Hernández et al., 2020

*B cell subset, peripheral blood B cell subset analyzed in the reported studies; BAL, bronchoalveolar lavage; (%), as frequency values; (#), as absolute number; Cases, COVID-19 patients (irrespective of severity); ICU, intensive care unit patients; Recovered, non-hospitalized subjects who had recovered from SARS-CoV-2 infection; HD, healthy donors; Outpatients, outpatients with milder disease; ARDS, acute respiratory distress syndrome; NCU, normal care unit patients; NCU (non-COVID-19), normal care unit patients treated for other reasons than COVID-19; ICU (non-COVID-19), intensive care unit patients treated for other reasons than COVID-19; Asymptomatic, patients who tested positive but had no symptoms.*

*^ρ^included subjects were in the following condition(s): high flow nasal cannula–noninvasive, ventilator (non-ARDS), mild to severe ARDS, and ECMO.*

*^γ^Patients with > 10L or 60% supplemental oxygen, managed in ICU.*

*^δ^Patients with < 3L or 28% supplemental oxygen.*

*^ε^Patients with < 10L or < 60% supplemental oxygen, requiring non-invasive ventilation (NIV) or continuous positive airway pressure (CPAP).*

*^ζ^Patients with clinical signs of pneumonia with one of the following: respiratory rate > 30 breaths/min, severe respiratory distress, or SpO_2_ < 90% on room air.*

*^η^Patients experiencing the following: respiratory failure, respiratory rate > 30 bpm, oxygen saturation < 92% at rest, arterial partial pressure of oxygen (PaO_2_)/fraction of inspired oxygen (FiO_2_) (PaO_2_/FiO_2_) ratio < 300 mmHg.*

*^θ^Fever, signs of airway disease, with or without a tomographic image indicating pneumonia.*

*^ι^Any of the following: requirement for mechanical ventilation, shock, or concomitant organ failure.*

*^κ^Respiratory distress, *i.e*., ≥ 30 breaths/min. in resting state, oxygen saturation of 90% or less on room air; or arterial partial pressure of oxygen (PaO_2_)/fraction of inspired oxygen (FiO_2_) of 300 or less.*

*^λ^Hospitalized patients.*

*^μ^Ambulatory patients.*

*^ν^Spleen hypofunction patients.*

*^ξ^Patients who underwent splenectomy for trauma.*

*^π^Patients requiring no hospitalization and experiencing symptoms like with fever, myalgia, and fatigue without obvious chest high resolution computed tomography findings for COVID-19.*

Only depletion of peripheral IgM+ memory B cells seems to be a hallmark of SARS-CoV-2 infection (Carsetti et al., 2020; Lenti et al., 2020). Intriguingly, the percentage of IgM+ and switched memory B cells showed an inverse correlation with symptoms duration in convalescent patients, with no age or gender related influence (Newell et al., 2021).

As expected in immunocompetent subjects, seroconversion is positively associated with frequency of switched memory B cells, IgM+ memory B cells, CD19+CD27hiIgM-IgD+ c-delta switched memory B cells, and CD19+ CD11c+Tbet+ ABCs, whose percentages are higher in patients with a high seroconversion index vs. patients with a low seroconversion index (Galbraith et al., 2021).

Late memory CD27-IgD- Double negative (DN) B cells are exhausted memory cells that actively contribute to the inflammatory status associated with aging by secreting TNF-α and IL-6 (Bulati et al., 2014; Crooke et al., 2019; Fraussen et al., 2019; Ma et al., 2019; Hagen and Derudder, 2020; Pietrobon et al., 2020).

DN B lymphocytes reached a statistically significant expansion in both COVID-19 patients and convalescent subjects vs. controls (Table 7; Mathew et al., 2020; Shuwa et al., 2021). However, at an individual patient level, DN B cell fraction tends to decrease from acute disease to convalescent status (Newell et al., 2021; Shuwa et al., 2021). A keen characterization of DN B cell subsets, revealed that the increase of DN B cells in severe and critical patients may be attributable to an augmented CD38–CD24–CD21–CD11c+ DN2 and CD38–/+CD24–CD21–CD11c– DN3 fraction (Table 7; Sosa-Hernández et al., 2020).

As regards SARS-CoV-2 specific memory, widening evidences demonstrated that generation of SARS-CoV-2 memory B cells initiates early after infection/symptoms onset and is durable (Hartley et al., 2020; Abayasingam et al., 2021; Byazrova et al., 2021; Carsetti et al., 2021; Dan et al., 2021; Gaebler et al., 2021; Long et al., 2021; Ogega et al., 2021; Sakharkar et al., 2021; Sherina et al., 2021; Sokal et al., 2021; Tong et al., 2021). It remains to be deepened if sex may play a role in influencing the time of onset of B cell memory in COVID-19 subjects.

### Plasma Blasts and Antibody Secreting Cells

Aging hampers the proper differentiation of B cells into plasma cells (Ventura et al., 2017; Crooke et al., 2019; Hazeldine and Lord, 2020). This phenomenon seems to be avoided during SARS-CoV-2 infection, since the plasmblast fraction of B cells was reported as augmented in all patients whereas levels tend to be restored in convalescent and recovered patients (Mathew et al., 2020; Sosa-Hernández et al., 2020; Long et al., 2021; Shuwa et al., 2021; Wildner et al., 2021; Table 8). This piece of data reflects an infection related reactive expansion, given that the percentage of plasma cells positively correlates with the fraction of IgG+ and IgA+ positive B cells (Shuwa et al., 2021). The increase in plasma cells showed a direct correlation with an oligoclonal expansion of antibody clones (Kuri-Cervantes et al., 2020). Reported evidences for COVID-19 seem to recall the contraction of the B cell repertoire which was demonstrated in older people, although the involvement of the same molecular mechanisms remains to be elucidated (Aiello et al., 2019; Crooke et al., 2019; Ma et al., 2019; Lian et al., 2020). Obviously, plasma cells exhibited a significant association with seroconversion, with frequencies being higher in patients with high seroconversion indices vs. those with low ones (Galbraith et al., 2021).

**TABLE 8:** Observed fluctuation in ASCs and plasmablasts in adult COVID-19 patients.

ASCs and plasmablasts	Observation	Analyzed patients	Compared group(s)	References
CD19+/–IgM–IgD–CD38+/+ ASCs	No change (%)	Severe^λ^	Mild^μ^	Ogega et al., 2021
	No change (%)	Severe^λ^	HD	Ogega et al., 2021
	No change (%)	Mild^μ^	HD	Ogega et al., 2021
CD19+CD21–CD27+CD38+/high ASCs	No change	Recovered^*ς*^	HD	Long et al., 2021
CD27hiCD38hi plasmblasts	Increase (%)	Severe^γ^	Convalescent	Shuwa et al., 2021
	Increase (%)	Severe^γ^	HD	Shuwa et al., 2021
	Increase (%)	Moderate^ε^	Convalescent	Shuwa et al., 2021
	Increase (%)	Moderate^ε^	HD	Shuwa et al., 2021
	Increase (%)	Mild^δ^	HD	Shuwa et al., 2021
	Increase (%)	Convalescent	HD	Shuwa et al., 2021
CD27+CD38+ plasmablasts	Increase (%)	Cases	Recovered^ρ^	Mathew et al., 2020
	Increase (%)	Cases	HD	Mathew et al., 2020
	Increase (%)	Cases	HD	Wildner et al., 2021;
	Increase (%)	ICU	Outpatients	Woodruff et al., 2020
	Increase (%)	ICU	HD	Woodruff et al., 2020
CD27+ CD38hi ASC/plasmablasts	Increase (%)	Severe^η^	HD	Sosa-Hernández et al., 2020
	Increase (%)	Critical^ι^	HD	Sosa-Hernández et al., 2020
	Increase (%)	Mild/moderate^θ^	HD	Sosa-Hernández et al., 2020
CD38+CD24– plasmablasts	Increase (%)	Severe^ζ^	Asymptomatic	Carsetti et al., 2020
	Increase (%)	Severe^ζ^	Mild^π^	Carsetti et al., 2020
	Increase (%)	Severe^ζ^	Contacts^σ^	Carsetti et al., 2020

*ASCs, antibody secreting cells; (%), as frequency values; Cases, COVID-19 patients (irrespective of severity); ICU, intensive care unit patients; HD, healthy donors; Outpatients, outpatients with milder disease; Asymptomatic, patients who tested positive but had no symptoms.*

*^γ^Patients with > 10 L or 60% supplemental oxygen, managed in ICU.*

*^δ^Patients with < 3 L or 28% supplemental oxygen.*

*^ε^Patients with < 10 L or <60% supplemental oxygen, requiring non-invasive ventilation (NIV) or continuous positive airway pressure (CPAP).*

*^ζ^Patients with clinical signs of pneumonia with one of the following: respiratory rate > 30 breaths/min, severe respiratory distress, or SpO_2_ < 90% on room air.*

*^η^Patients experiencing the following: respiratory failure, respiratory rate > 30 bpm, oxygen saturation < 92% at rest, arterial partial pressure of oxygen (PaO_2_)/fraction of inspired oxygen (FiO_2_) (PaO_2_/FiO_2_) ratio < 300 mmHg.*

*^θ^Fever, signs of airway disease, with or without a tomographic image indicating pneumonia.*

*^ι^Any of the following: requirement for mechanical ventilation, shock, or concomitant organ failure.*

*^κ^Respiratory distress, i.e., ≥30 breaths/min. in resting state, oxygen saturation of 90% or less on room air; or arterial partial pressure of oxygen (PaO_2_)/fraction of inspired oxygen (FiO_2_) of 300 or less.*

*^λ^Hospitalized patients.*

*^μ^Ambulatory patients.*

*^ν^Spleen hypofunction patients.*

*^ξ^Patients who underwent splenectomy for trauma.*

*^π^Patients requiring no hospitalization and experiencing symptoms like with fever, myalgia, and fatigue without obvious chest high resolution computed tomography findings for COVID-19.*

*^ρ^Non-hospitalized subjects who had recovered from SARS-CoV-2 infection.*

*^*ς*^Adults with a prior positive COVID-19 PCR test who met the definition of recovery based on the guideline from the Chinese Center for Disease Control and Prevention.*

*^σ^Contacts of SARS-CoV-2 confirmed cases who were negative by qPCR.*

Similar results were replicated at the lung level. In fact, the total number and proportion of BAL plasma cells was increased in COVID-19 patients (according with disease severity) vs. healthy controls (Kim C.W. et al., 2021).

### B Cell Trafficking Phenotype

B cell trafficking phenotype exhibits profound modification during aging (Bulati et al., 2014).

During acute COVID-19, B cells showed a reduced expression of CXCR3, CXCR5 and integrin β7 according to disease severity, with CXCR3 and CXCR5 level normalization in convalescent subjects (Shuwa et al., 2021).

With a detailed characterization of B cell subsets, it was documented that CXCR5 expression was reduced on naïve, plasmablasts, and memory B cells, including DN memory B cells (Kuri-Cervantes et al., 2020; Mathew et al., 2020). This is a very intriguing piece of data, since CXCR5 did not show any statistically significant difference in its expression on B lymphocyte subsets comparing young and old subjects (Bulati et al., 2014), thus it is not expected to be differentially modulated in COVID-19 as a consequence of aging.

BAL B lymphocytes from patients with mild disease express CCR6 and CXCR3, whereas BAL B cells from patients with severe disease express more CXCR4 vs patients with mild disease and healthy subjects (Kim C.W. et al., 2021). Given that both CCR6 and CXCR4 are involved in B cell circulation to lymph nodes, whereas CXCR3 (whose gene is located on X chromosome)^1^ rules B cell attraction to inflammation sites (Kunkel and Butcher, 2003), it would be worth deepening if BAL B cells from COVID-19 exhibit different B cell trafficking features according to both severity and gender. Also, in severe patients, BAL plasma cells had an increased expression of CCR2 and CCR10 (Kim C.W. et al., 2021). CCR2 is normally expressed on normal plasma cells, is renowned for mediating homing aberrant plasma cells in multiple myeloma and is downregulated during B cell maturation, while CCR10 is highly expressed by IgA producing plasma cells (Vande Broek et al., 2003; Flaishon et al., 2004; Shirakawa et al., 2008; Hu et al., 2011). Also, in terminally differentiating B cells CCR10 expression may be induced by 1,25-Dihydroxyvitamin D3, a known modulator of B cell homeostasis that showed a correlation with COVID-19 severity (Martens et al., 2020; Notz et al., 2021).

### Functional Considerations About B Cells

As previously mentioned, inflammageing -defined as the balance between inflammatory and anti-inflammatory mechanisms pending toward inflammation- is a typical hallmark of an aging immune system (Ventura et al., 2017; Hazeldine and Lord, 2020).

COVID-19 offers the chance to improve the knowledge about the deregulation of molecular mechanisms ruling the production of inflammatory mediators. In fact, cytokine storm, characterized by increased circulating cytokine levels, with potential life-threatening acute systemic inflammation and secondary organ dysfunction, is a prominent feature of SARS-CoV-2 infection especially in severe cases (see section “Cytokine storm in COVID-19 disease”).

As expected, after TLR9 agonist CpGB stimulation the percentage of IL-6+ B cells increased in acute COVID-19 patients vs. healthy controls and convalescent patients irrespective of possible chest X-ray abnormality (Shuwa et al., 2021). On the contrary, the percentage of IL-10+ B cells was significantly lower in patients with persistent lung pathology vs. healthy controls and normal chest X-ray subjects (Shuwa et al., 2021). However, analyzed at an individual patient level, the frequency of IL-10+ B cells increased from acute phase to convalescent status (Shuwa et al., 2021).

Transcriptomic analysis revealed that B cell immune answers are predominant in BAL fluid (Cavalli et al., 2020), and that BAL B cells from severe COVID-19 patients were highly activated by TNF-α signaling (Kim C.W. et al., 2021). B-cell activating factor (BAFF) receptor and transmembrane activator and calcium modulator and cyclophilin ligand interactor were expressed by all patients, but in severe patients there was an upregulation of apoptosis related gene transcription, probably mediated by BAFF signaling (Kim C.W. et al., 2021).

Study of peripheral B lymphocytes revealed a pattern of activation/exhaustion, characterized by increased frequency of CD69 and CD95 in hospitalized subjects vs. healthy controls and convalescent patients, and by an increase in PD1 frequency in non-hospitalized (convalescent) individuals vs. both healthy controls and COVID-19 hospitalized patients (Files et al., 2021). This may mirror the establishment of efficient mechanisms modulating strength and durability of B cell responses in convalescent subjects, given that both CD69 and CD95 are markers of lymphocyte activation (Catlett and Bishop, 1999; Vazquez et al., 2009), whereas PD1 is an immune modulator (Wang et al., 2019). Also, CD95 expression is increased on CD27+ B cells from older individuals vs. younger donors (Chong et al., 2005), and might be involved in age related differences in the regulation of anti-SARS-CoV-2 immune responses.

## Mesenchymal Stem Cell Transplantation in COVID-19 Patients: Rationale and Results

Mesenchymal stem cells, also known as mesenchymal stromal cells (MSCs) (Viswanathan et al., 2019), are heterogeneously multipotent stem cells that can be isolated from a variety of sources, including umbilical cord, human tissues like bone marrow and adipose tissue (using the stromal vascular fraction – SVF), and menstrual blood (Blaber et al., 2012; Kallmeyer and Pepper, 2015; Elgaz et al., 2019; Copcu, 2020; Gentile and Sterodimas, 2020a,b; Jeyaraman et al., 2020; Juárez-Navarro et al., 2020; Lin et al., 2020; Barros et al., 2021; Gentile, 2021; Mazini et al., 2021; Xu et al., 2021).

Mesenchymal stem cells are universally renowned for their unique immunomodulatory properties; once led into circulation, they are able to reach inflammation sites (Kallmeyer and Pepper, 2015), where they exert an inhibitory function on neutrophils, monocytes, dendritic cells, NK, B and T lymphocytes (Elgaz et al., 2019; Juárez-Navarro et al., 2020; Kim H. et al., 2020; Moradinasab et al., 2021). MSC ruled immunomodulation is performed by direct cell–cell interaction (through the expression of molecules like B7H1, PD-L1, and PD-L2) (Elgaz et al., 2019; Lin et al., 2020) as well as by a paracrine action, mediated by vehiculation of anti-inflammatory mediators through extracellular vesicles (Schulman et al., 2018; Martin-Rufino et al., 2019; Gowen et al., 2020; Juárez-Navarro et al., 2020; Kim H. et al., 2020; Lin et al., 2020; O’Driscoll, 2020; Wang J. et al., 2020; Gentile, 2021; Raghav et al., 2021; Su et al., 2021) and by cytokine (IL-10, transforming growth factor-β, TNF-stimulated gene 6 protein, IFN-γ) and soluble factor indoleamine-pyrrole 2,3-dioxygenase, prostaglandinE2, nitric oxide) secretion (Schulman et al., 2018; Elgaz et al., 2019; Martin-Rufino et al., 2019; Juárez-Navarro et al., 2020; Lin et al., 2020; Shetty, 2020; Gentile, 2021; Raghav et al., 2021). MSC polarization toward an anti-inflammatory phenotype is exacerbated by TLR stimulation elicited by pathogen components, like viral RNA (Waterman et al., 2010). Also, MSCs retain a differentiation potential making them ideal contributors to tissue repair (Blaber et al., 2012; Kallmeyer and Pepper, 2015; Elgaz et al., 2019; Copcu, 2020; Jeyaraman et al., 2020; Juárez-Navarro et al., 2020; Rogers et al., 2020).

As suggested by successful MSC use in a number of pathological scenarios involving uncontrolled immune activation with consequent tissue damage, like for example H9N2 induced acute lung injury, ARDS, autoimmune diseases and graft-versus-host disease (Waterman et al., 2010; Elgaz et al., 2019; Coelho et al., 2020; Yen et al., 2020; Barros et al., 2021; Musial and Gorska-Ponikowska, 2021; Su et al., 2021), the undeniable immunomodulatory and inflammation relieving properties of MSCs make them putative candidates as rejuvenating factors for the older immune system, on the basis of the documented ability to reduce pro-inflammatory mediators and to promote the expansion of regulatory lymphocyte subsets (Yeo et al., 2021). Also, MSC transplantation can contribute to the amelioration of aging frailty (Schulman et al., 2018; Florea et al., 2019; Sun et al., 2019), but MSC may exhibit age related deteriorating functions linked to inflammageing, especially in terms of alterations in number and characteristics of extracellular vesicles, DAMP production, excessive IL-6 release, loss of MSC ability to shift monocyte polarization toward M2, and triggering of ineffective hemopoiesis (Tsuruhara et al., 2017; Lee and Yu, 2020). Potency also seems to be affected by aging in bone marrow derived MSCs (BM-MSCs), but not in MSC obtained from adipose tissue (Rogers et al., 2020).

Assuming also that MSCs are negative for ACE2 and TMPRSS2 SARS-CoV-2 receptor complex (Leng et al., 2020; Moradinasab et al., 2021), MSC transplantation or infusion of MSC derived extracellular vesicles in the context of a deregulated immune response, cytokine storm and lung injury elicited during COVID-19 (especially in older people) appeared as a promising strategy (Gentile and Sterodimas, 2020a,b; Gorman et al., 2020; Monguió-Tortajada et al., 2020; Qin and Zhao, 2020; Rogers et al., 2020; Tsuchiya et al., 2020; Gentile, 2021; Raza et al., 2021).

Following the first paper by Leng et al. (2020) (see below), a number of data extrapolated from ongoing and concluded clinical studies on COVID-19 patients using both BM-MSCs and umbilical cord MSCs (UC-MSCs), together with reports employing MSCs from other sources or MSC vesicles, with positive results have been published so far (Al-Khawaga and Abdelalim, 2020; Barkama et al., 2020; Gentile et al., 2020; Golchin et al., 2020; Gorman et al., 2020; Liang et al., 2020; Lin et al., 2020; Meng F. et al., 2020; Sánchez-Guijo et al., 2020; Sengupta et al., 2020; Shu et al., 2020; Gentile, 2021; Jamshidi et al., 2021; Kouroupis et al., 2021; Moradinasab et al., 2021; Payares-Herrera et al., 2021; Sharma and Zhao, 2021).

In that paper, Leng et al. (2020) showed that MSCs cured or significantly improved the functional outcomes of seven patients without observed adverse effects. After treatment, the peripheral lymphocytes were increased, the C-reactive protein as well as TNF-α decreased, while IL-10 increased, and the overactivated cytokine-secreting immune cells CXCR3+CD4+ T cells, CXCR3+CD8+ T cells, and CXCR3+ NK cells disappeared in 3–6 days. In addition, a group of CD14+CD11c+CD11bmid regulatory DC cell population dramatically increased. Therefore, the intravenous transplantation of MSCs was safe and effective for treatment in patients with COVID-19 pneumonia, especially for patients in critically severe condition.

It was then demonstrated that both BM- and UC-MSCs showed to be able to improve COVID-19 survival rates (Shu et al., 2020; Häberle et al., 2021; Lanzoni et al., 2021; Moradinasab et al., 2021), symptoms (Liang et al., 2020; Meng F. et al., 2020; Shu et al., 2020; Tang et al., 2020; Zengin et al., 2020; Hashemian et al., 2021; Moradinasab et al., 2021), pulmonary functions (Liang et al., 2020; Meng F. et al., 2020; Shu et al., 2020; Zengin et al., 2020; Häberle et al., 2021; Moradinasab et al., 2021), inflammatory marker levels (CRP, TNF-α, neutrophil extracellular traps, IL-1RA, IL-5, IL-6, IL-18, IL-27, IL-17E/IL-25, IL-17F, CXCL-1, neutrophil-to-lymphocyte ratio, D-dimer) (Guo Z. et al., 2020; Liang et al., 2020; Tang et al., 2020; Zengin et al., 2020; Zhang Y. et al., 2020; Hashemian et al., 2021; Lanzoni et al., 2021; Moradinasab et al., 2021) and lymphopenia (Guo Z. et al., 2020; Liang et al., 2020; Zhu Y. et al., 2020) after few days of treatment. Placenta derived MSCs (Barkama et al., 2020), adipose derived MSCs (Sánchez-Guijo et al., 2020) and menstrual blood derived MSCs (Xu et al., 2021) showed to have similar effects.

In the context of the possible therapeutic use of MSCs to promote lung regeneration in COVID19, adipose and bone marrow derived MSCs are able to contribute to lung regeneration in animal models, by differentiation into type 2 alveolar epithelial cells and their immunomodulatory potential; in the case of adipose derived MSC, the success of both mechanisms is facilitated by their large availability (Copcu, 2020; Behnke et al., 2020; Gentile and Sterodimas, 2020a,b; Rogers et al., 2020). The same regenerative properties are shown by SVF, whose cellular components secrete pro-angiogenetic and immune-modulating mediators (Gentile and Sterodimas, 2020a,b; Gentile, 2021).

As regards the preference for autologous rather than allogenic alternatives, the choice is influenced by limited time availability as well as intrinsic dependence of MSC properties on the age of the donor; in fact, given that also MSC experience senescence (Lee and Yu, 2020), the choice of allogenic MSCs or more senescence resistant adipose derived MSC may be recommendable in older patients (Rogers et al., 2020; Moradinasab et al., 2021).

Molecular mechanisms triggered by MSC infusion and by exosome derived MSCs in COVID-19 patients are currently under investigation. As regards immune cell homeostasis, overactivated cytokine-secreting immune cells CXCR3+CD4+ T cells, CXCR3+CD8+ T cells, and CXCR3+ NK cells disappeared soon after the MSC transplantation (Leng et al., 2020), with a general increase in both percentage and count of B lymphocytes, NK, CD3+, CD4+ and CD8+ cells (Liang et al., 2020; Sengupta et al., 2020; Tang et al., 2020; Zhang Y. et al., 2020; Zhu Y. et al., 2020) up to the decrease of neutrophil-to-lymphocyte ratio (Liang et al., 2020). This is nice food for thought, since MSC immunomodulation activity is exerted through inhibition of B, CD4+ and CD8+ T cell proliferation (Budoni et al., 2013; Joel et al., 2019; Moradinasab et al., 2021). Instead, MSC action on neutrophils is mainly functional rather than “numerical” (Li and Hua, 2017; Joel et al., 2019; Lin et al., 2020; Moradinasab et al., 2021), but the available data demonstrate that in more severe COVID-19 cases and in deceased patients neutrophils are also more abundant other that involved in cytokine storm establishment (see sections “Introduction” and “Neutrophils”). It remains to be demonstrated that MSC elicited an increase in COVID-19 T and B cell numbers may be related to an increase in the proportion of Tregs or Bregs, as observed in other contexts (Joel et al., 2019; Liu J. et al., 2020; Moradinasab et al., 2021) or if the apoptotic rate in neutrophils may be increased specifically in MSC treated patients. MSCs hamper B cell differentiation into plasma cells (Tsuruhara et al., 2017; Moradinasab et al., 2021); the same effect on COVID-19 patients and eventual consequences on anti-SARS-CoV-2 antibody production deserve further investigation. In addition, MSCs alter B cell trafficking phenotype, including downregulation of CXCR4 and CXCR5 (Joel et al., 2019), that are differentially regulated in COVID-19 (see section “B Cell Trafficking Phenotype section”). Molecular changes elicited by MSC influencing recirculation of B cells to lungs in the case of SARS-CoV-2 infection are still unexplored. Finally, given DN B cell increase in both immunosenescence and COVID-19 (see section “Memory B cells”), the potential rejuvenating effect of MSC on the exhausted subset, like DN B cells, might represent a putative mechanism of action contributing to the amelioration of these inflammatory conditions.

Similarly, (as mentioned above) CD14+CD11c+CD11bmid regulatory DC cell population increased after MSC transplantation, (Leng et al., 2020). The origin of CD14+CD11c+CD11bmid regulatory DC cells deserve to be assessed, because (I) CD11c+ DCs are reported as reduced in COVID-19 patients and show multiple functional alterations [see section “Dendritic cells (DCs)”] (II) the exploration of such an ontological pattern would put some more light on monocyte dynamics. CD11c is highly expressed by conventional DC2, powerful stimulators of naïve T cells (Rhodes et al., 2019) reduced in COVID-19 [see section “Dendritic cells (DCs)”] and by monocyte derived DCs (Delirezh et al., 2011; Boyette et al., 2017; Rhodes et al., 2019; Chometon et al., 2020). Despite this detail, no functional defects in conventional DC2 or monocyte derived DCs generation were studied in SARS-CoV-2 infected individuals so far. Data for better IL-1β, IL-6, and TNF-α producer classical macrophages (CD16-) are instead more commonly settled on detecting no variation in their frequency (see section “Monocytes and macrophages”). Since all monocytes are able to differentiate into macrophages, but (I) classical monocytes have a superior ability to differentiate into monocyte derived DCs (expressing CD11c) and (II) the relationship between conventional DC2 and monocytes is controversial (Delirezh et al., 2011; Boyette et al., 2017; Rhodes et al., 2019; Chometon et al., 2020), it would be worth discovering what molecular pathways are activated in classical monocytes by interacting with MSCs that may influence their immunophenotypic and functional faith.

Also, DCs exhibit a number of functional alterations in COVID-19, including reduced expression of CD86 and HLA-DR, whose upregulation is specifically hampered by MSCs during DC maturation (Joel et al., 2019). It remains to be assessed if this effect of MSCs on DCs is summed to that elicited by the microenvironment in SARS-CoV-2 infected tissue.

Together, these results underscore the role of MSCs in improving COVID-19 patient outcomes via maintenance of immune homeostasis, i.e., improving immunosenescence and relieving immunopathology.

## Conclusion

**FIGURE 1:**
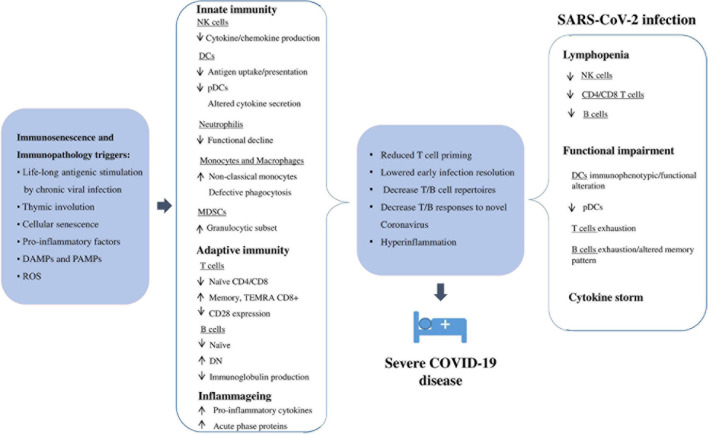
A schematic view of how immunopathology and immunosenescence could contribute to severe COVID-19 in older people. PAMPs, pathogen associated molecular patterns; ROS, reactive oxygen species. See text for other acronyms.

Summarizing clinical findings discussed in the present paper, it is clear that the synergistic effects of immunosenescence and inflammageing (i.e., immunopathology) in older individuals have an important impact on their immune responses to SARS-CoV-2 infection (Figure 1) and should be taken into account whenever looking for factors influencing mortality rates in COVID-19 (Salimi and Hamlyn, 2020; Bajaj et al., 2021; Chen Y. et al., 2021). Changes in innate immune responses and the failure to trigger an effective acquired immune response (i.e., immunosenescence), in combination with a higher pro-inflammatory status (i.e., immunopathology) should explain why older people do not appropriately control viral replication and the potential clinical consequences triggered by a cytokine storm, also reducing the chances of proper recovery after infection resolution (Salimi and Hamlyn, 2020; Bajaj et al., 2021; Chen Y. et al., 2021). This awareness is critical to the implementation of any strategy aimed at improving protective immunity and vaccine efficacy against SARS-CoV-2 in the older population (Bajaj et al., 2021). Regarding the gender differences in SARS-CoV-2 infection outcome reported in the present review, most studies are observational only and do not take into account that males and females may have several pre-existing conditions affecting the chance of successful aging, hence involving different responses to virus infection (Leng and Margolick, 2020; Mauvais-Jarvis et al., 2020; Sampathkumar et al., 2020; Ligotti et al., 2021; Lio et al., 2021). The key roles of immunosenescence and immunopathology in the outcome of SARS-CoV-2 infection are further supported by the beneficial results obtained with MSC infusion that, as previously discussed, act restoring immune homeostasis and contributing to lung repair (Gentile and Sterodimas, 2020a, b; Gorman et al., 2020; Monguió-Tortajada et al., 2020; Qin and Zhao, 2020; Rogers et al., 2020; Tsuchiya et al., 2020; Gentile, 2021; Raza et al., 2021). Therefore, the enhancement of the efficacy of the acquired immune response and the relief of the pro-inflammatory status should be an important issue both for SARS-CoV-2 infection resolution as well as for the appropriate generation of immunity upon vaccination.

## Author Contributions

All the authors contributed to draft the manuscript, revised the manuscript, and approved the final version. ML, FP, and CC wrote the final version.

## Conflict of Interest

The authors declare that the research was conducted in the absence of any commercial or financial relationships that could be construed as a potential conflict of interest.

## Publisher’s Note

All claims expressed in this article are solely those of the authors and do not necessarily represent those of their affiliated organizations, or those of the publisher, the editors and the reviewers. Any product that may be evaluated in this article, or claim that may be made by its manufacturer, is not guaranteed or endorsed by the publisher.

## Footnotes

1 https://www.genecards.org/cgi-bin/carddisp.pl?gene=CXCR3&keywords=cxcr3 (accessed July 22, 2021).
